# Bioinspired Adaptive-Depth Neural Growth for Deepfake Video Forensics: An Entropy-Guided State-Space Framework

**DOI:** 10.3390/biomimetics11090653

**Published:** 2026-09-11

**Authors:** Muhammad Hussain, Fahman Saeed, Sultan Aldera

**Affiliations:** 1 Computer Science Department, King Saud University, Riyadh 11451, Saudi Arabia; 2 Computer Science Department, College of Computer and Information Sciences, Imam Mohammad Ibn Saud Islamic University (IMSIU), Riyadh 11432, Saudi Arabia; faesaeed@imamu.edu.sa (F.S.); ssaldera@imamu.edu.sa (S.A.)

**Keywords:** deepfake detection, Celeb-DF v2, state-space model, selective SSM, Mamba, FastICA, Sinkhorn normalization, adaptive depth growth, video forensics, temporal modeling, progressive training

## Abstract

Deepfake videos currently facilitate extensive financial deception, political misinformation, and unauthorized imagery, with anticipated U.S. losses from deepfake-related fraud surpassing $40 billion by 2027; human evaluators accurately recognize high-quality forgeries merely 25% of the time, highlighting the pressing necessity for automated, widely applicable detection mechanisms. Adaptive-depth architectures offer an intriguing alternative to fixed-depth deepfake detectors when the optimal model capacity is indeterminate in advance. This study presents the Adaptive Entropy-Guided ICA State-Space Model Forgery Detector (AEGIS-FD), a deepfake detection framework at the video level that progressively increases its depth from one to eight layers via an entropy-driven growth mechanism, attaining peak validation performance at a depth of six. The design incorporates a three-dimensional spatiotemporal stem, Sinkhorn-normalized manifold-constrained hyper-coupling (mHC) layers for balanced temporal integration, a selected state-space temporal block for sequence depiction, and FastICA-based initialization for newly introduced layers. Evaluated using Celeb-DF v2, AEGIS-FD achieves a test AUC of 0.9600 and a validation AUC of 0.9607, above the performance of a single-layer Mamba SSM baseline (AUC = 0.8355). In comparison to a fixed-depth-6 baseline, the model demonstrates consistent improvements over five random seeds (96.12 ± 0.28 vs. 94.84 ± 0.44 AUC; *p* = 0.0022), suggesting that adaptive development provides advantages that exceed mere depth. In a zero-shot cross-dataset evaluation—trained on Celeb-DF v2 and assessed without fine-tuning on a FaceForensics++ (FF++) C23 subset comprising 1000 original and 1000 FaceSwap videos—AEGIS-FD achieves an AUC of 89.2 compared to 88.4 for the corresponding baseline (+0.8 AUC), providing initial proof of cross-dataset transferability. These findings suggest that adaptive-depth growth presents a viable approach for detecting deepfakes at the video level, while further validation across various datasets and modification techniques is essential.

## 1. Introduction

The convergence of generative adversarial networks (GANs), variational autoencoders (VAEs), and contemporary diffusion models has substantially lowered the barrier to making hyper-realistic synthetic face videos [1]. Deepfakes—artificially created or edited face videos—have advanced from crude face swap artifacts to flawless, identity-preserving modifications indistinguishable from original footage by the naked human eye [2]. Empirical research demonstrates that human viewers can reliably recognize high-quality synthetic films about 24.5% of the time, whereas over 68% of current deepfakes are visually indistinguishable from genuine media [3]. The world is currently experiencing an unprecedented spike in generative AI fraud, with deepfake material estimated to jump from 500,000 files in 2023 to 8 million by 2025, a reported 900% annual growth rate. This “viral proliferation” is driven by cheap, convincing methods like voice cloning and has already prompted a 3000% surge in fraud attempts internationally. With human detection rates as low as 24.5% for high-quality fakes, the financial stakes are immense, with U.S. losses alone predicted to top $40 billion by 2027 [4].

Beyond financial fraud, deepfakes are exploited for political disinformation, non-consensual intimate imagery (accounting for 96–98% of all deepfakes online), identity theft, and legal evidence manipulation. A 2024 McAfee survey indicated that 32% of individuals had grown much more distrustful of social media content, while 63% of Americans claim doubt about factual events owing to changed footage [5]. UNESCO’s 2025 report notes a “liar’s dividend” in which the sheer presence of deepfake technology enables bad actors to discard legitimate recordings as probable fakes. These findings collectively underline that automatic, scalable, and generalizable deepfake detection is not only a computer vision task but a fundamental infrastructural problem for modern democratic society [6].

The research community has responded with an expanding ecosystem of detection methods, benchmarks, and datasets. Early convolutional neural network (CNN)-based frame-level detectors such as Xception and MesoNet achieved near perfect accuracy on FaceForensics++ but generalized poorly to unknown manipulation types and higher quality synthesis pipelines [7]. The introduction of Celeb DF at CVPR 2020 highlighted a large generalization gap: the nine top pretrained detectors scored a mean AUC of only roughly 56.9% on Celeb DF, compared to over 80% on earlier standards [8]. This disparity is caused by Celeb DF’s improved synthesis pipeline, which reduces coarse blending artifacts, normalizes skin color, and delivers temporally coherent identity-preserving alterations. More contemporary technologies, such as transformer-based video detectors and frequency domain systems, have improved detection rates but come with significant computing overhead or restricted temporal modeling capability.

A persistent architectural constraint across existing detectors is the use of static, fixed-depth networks whose depth is established before training begins. In practice, the ideal depth depends on dataset complexity, class imbalance, and the distribution of forging artifacts, all of which represent significant challenges in machine learning [8]. Shallow fixed-depth networks underfit sophisticated temporal connections, whereas excessively deep networks overfit on the meager authentic video data provided in Celeb DF v2 (only 890 real movies compared to 5639 fraudulent). Adaptive architectural growth strategies where depth extends progressively in response to training dynamics offer a logical alternative to static design but remain underexplored in video deepfake detection.

Notwithstanding these advancements, a pivotal unresolved inquiry persists: does entropy-guided adaptive-depth growth enhance generalization relative to fixed-depth architectures of equivalent capacity, or are the observed improvements predominantly attributable to a higher parameter count? This study explicitly examines this inquiry via matched fixed-depth baselines and cross-dataset assessment, while also presenting the AEGIS-FD architecture. An additional obstacle pertains to the redundancy of features in temporal representations. When feature channels transmit overlapping or related information, incorporating additional layers results in reduced benefits. Regulating feature mixing entropy by a logical normalization method, particularly the Sinkhorn–Knopp algorithm for the creation of doubly stochastic matrices, offers a theoretically grounded approach to reallocating channel information and assessing the necessity for additional capacity. Moreover, initializing freshly created layers with statistically independent components derived from FastICA guarantees that new modules commence from a varied, disentangled feature base instead of an arbitrary beginning position.

Our primary quantitative results are as follows: AEGIS-FD achieves a test AUC of 0.9600 on Celeb-DF v2, surpassing a comparable fixed-depth-6 baseline by an average of +1.28 AUC across five random seeds (96.12 ± 0.28 compared to 94.84 ± 0.44; paired *t*-test *p* = 0.0022), and demonstrates zero-shot transfer to a FaceForensics++ C23 subset with an AUC of 89.2 against 88.4 for the matched baseline.

This article presents the Adaptive Entropy-Guided ICA SSM Forgery Detector (AEGIS-FD), a methodology for detecting deepfakes at the video level specifically designed for Celeb-DF v2. Its primary contribution is conceptual instead of solely architectural: we introduce adaptive-depth growth as a realistic training framework for video deepfake forensics and present controlled empirical evidence indicating that an information-theoretic growth signal offers generalization advantages surpassing those of a fixed-depth capacity. (1) We propose an entropy-driven growth mechanism where the mixing entropy of Sinkhorn-normalized mHC layers, in conjunction with validation-AUC dynamics, determines whether the depth escalates from one to eight layers, employing a validation-gated retention test to eliminate expansions that fail to enhance generalization; (2) we present FastICA-based initialization for newly incorporated layers, linking growth to the statistics of existing feature representations, ensuring that each new module commences from a disentangled, data-informed foundation; (3) we implement the supporting architecture—a 3D spatiotemporal stem, mHC temporal blending layers, and a selective state-space temporal block at O(T·d^2^) cost, which is linear with respect to sequence length. Training and in-domain evaluation uses Celeb-DF v2; external zero-shot transfer is assessed on a specific segment of the FF++ C23 test division.

## 2. Related Work

### 2.1. Deepfake Generation Techniques

Understanding detection requires experience with generation. Face manipulations fall into four categories: complete face synthesis, identity switch (face swapping), facial attribute modification, and facial expression reenactment. Early face swapping, popularized under the term “DeepFake” on online sites, is based on a shared encoder dual decoder autoencoder architecture trained separately on source and target identities. To construct a false frame, the shared encoder and target decoder are applied to a source face, and the output is blended into the target scene by Poisson image editing [9].

Modern face swapping architectures, such SimSwap and FSGAN, leverage GAN-based refiners conditioned on facial identity vectors acquired by recognition networks, enabling unrestricted identity transfer with retained expression and gaze. Conditional GANs conditioned on facial landmarks, audio-driven mouth shapes, or posture vectors enable face recreation where the target identity’s expressions are replaced by a driving source [10]. StyleGAN and its derivatives power full face synthesis systems capable of constructing entirely nonexistent identities with photographic realism. Most recently, diffusion model-based generation has evolved as an alternative, yielding temporally smoother frames with fewer spectral GAN fingerprints [4,10]. This development from artifact-rich encoder decoder techniques to practically artifact free neural rendering clearly supports the need for temporal and feature-level detection beyond simple artifact spotting.

Celeb DF v2 [8] notably uses an enhanced encoder decoder face swap pipeline that corrects the color mismatch, temporal flickering, and geometric misalignment observed in prior datasets. The synthesis quality is comparable to videos circulating on public video platforms, making it the most challenging identity swap benchmark for testing generalizable detectors.

### 2.2. Deepfake Detection Benchmarks

The landscape of deepfake detection research has been shaped by successive generations of increasingly difficult benchmarks. UADFV was among the first video-level datasets that mostly examined eye blinking inconsistencies [11]. FaceForensics++ (FF++) standardized evaluation across four alteration methods—DeepFake, Face2Face, FaceSwap, and NeuralTextures—at several JPEG compression settings, enabling controlled research of detection under compression deterioration. Detectors trained and assessed on FF++ dataset (intra-dataset) achieved 90–100% accuracy, but exhibited poor transferability to other datasets.

The DeepFake Detection Challenge (DFDC) provided a large-scale real-world benchmark with actor-recorded movies and different capture settings. However, its manipulation quality varied greatly. Celeb DF v2 offers a qualitative leap: it features 590 genuine celebrity videos sourced from YouTube across varied ages, genders, and ethnicities, 300 additional YouTube real videos, and 5639 high-quality synthetic videos. Cross-dataset studies reveal serious generalization failures: CORE obtains 0.961 AUC on UADFV but only 0.741 on Celeb DF v2; FFD declines from 0.950 to 0.687; and even the best-performing model examined in the original benchmark scored just 65.5% AUC. In an attempt to address this gap, CrossDF more recently developed a Deep Information Decomposition (DID) framework that uses adversarial decorrelation learning to separate deepfake-relevant features from identity- and technique-specific variations. This improves generalization to unseen forgery methods without requiring target-domain fine-tuning [12]. These results support Celeb DF v2 as the primary stress test for generalizable deepfake identification.

More recent benchmarks such WildDeepfake, DF40, and DeepFake Eval 2024 further challenge detectors with diffusion model-generated content showing AUC drops of 45–50% for state-of-the-art models confronted with contemporary forgeries compared to legacy benchmarks [13]. DeepfakeBench presents a uniform evaluation process spanning 40 manipulation techniques, revealing that no single architecture yet delivers robust cross-dataset performance [13].

### 2.3. Frame-Level CNN Detection

The initial deep learning detectors handled deepfake detection as a per-frame binary image classification task. Rossler et al. proved that fine tuning Xception on FF++ provided near perfect intra-dataset accuracy but failed to generalize. MesoNet suggested a more compact architecture targeting mesoscopic facial properties texture statistics at intermediate spatial scales and showed that architectural design customized to deepfake artifacts was preferable to generic ImageNet pretrained backbones [14].

DSP FWA exploited the spatial pyramid of frequency artifacts created by face warping during the synthesis process. While effective for early pipelines, these spectral characteristics disappear in newer neural rendering techniques and in JPEG compressed outputs [15]. EfficientNet-based detectors have obtained greater accuracy on Celeb DF through aggressive face cropping using MTCNN or MediaPipe, multi-scale feature aggregation, and fine-grained feature comparison. For example, a SADFFD model combining EfficientNet B4 with multi SAM branches obtained 98.65% accuracy on Celeb DF by utilizing fine-grained facial area features, while this result uses full face cropping and identity-preserving preprocessing that introduces potential identity leakage in the split [16].

A major drawback of all frame-level techniques is their inability to use temporal consistency cues. Deepfake generators typically operate frame by frame, disrupting the natural temporal continuity of face identification traits, subtle motion dynamics, and temporal frequency patterns signals that are present only at the video level but are imperceptible in isolated frame analysis.

### 2.4. Temporal and Video Level Detection

Temporal modeling approaches explicitly incorporate multi-frame information. CNN LSTM hybrids integrate spatial feature extraction from EfficientNet or ResNet backbones with LSTM or GRU units to describe sequential dependencies across sampling frames. These architectures enhanced generalization on Celeb DF but still rely on frame-independent spatial characteristics that may miss temporally coherent but spatially subtle aberrations. 3D convolutional networks including TD 3DCNN and 3D attentional inception networks jointly represent appearance and short range motion through volumetric kernels, producing competitive results while suffering cubic parameter increase with increasing spatial resolution [17].

Transformer-based video models, including TimeSformer, ViViT, and Cross ViT, employ multi-head self-attention across both spatial tokens and temporal frames, attaining great performance on large-scale video understanding benchmarks. Applied to deepfake detection, Zheng et al. [18] introduced a transformer-based technique that explicitly models fine-grained facial areas throughout time, attaining competitive performance on Celeb DF v2. A recently proposed TSOM transformer addresses manipulation texture, shape, and ordering [19]. FatFormer (CVPR 2024) combines CLIP’s vision language space with forgery-aware attention and discrete wavelet transform frequency features [20]. D^3^ (CVPR 2025) scales up to multiple generators and employs a parallel discrepancy branch to learn universal artifacts [21].

Despite these advances, transformer-based video detectors carry quadratic computing complexity in sequence length and sometimes require massive pretraining corpora to generalize successfully. Pixelwise temporal frequency detection solves this using a 1D Fourier transform along the temporal axis for each pixel, recognizing temporal abnormalities in specified places via an attention suggestion module [22]. Spatiotemporal consistency approaches merge texture improvement blocks, spatial artifact attention, and temporal inconsistency attention, creating a more modular architecture. These methods together indicate that temporal modeling at different scales is crucial for robust detection on high-quality benchmarks.

### 2.5. State-Space Models for Video Understanding

State-Space Models (SSMs) have recently developed as a computationally efficient alternative to transformer self-attention for sequential data. The Mamba architecture introduces selective state spaces with input-dependent parameters Δ, B, and C, enabling content-based selective propagation and suppression while maintaining linear time complexity via a hardware-aware parallel scan. Mamba achieves 5× greater inference throughput than analogous transformers, with performance matching or exceeding transformers at 2× the parameter count on language modeling benchmarks [23].

VideoMamba (ECCV 2024) adapts Mamba to the video domain, demonstrating linear complexity long-term video modeling that beats 3D CNNs and video transformers on activity recognition benchmarks [24]. The Video Mamba Suite thoroughly analyzes SSMs across 14 model configurations and 12 video understanding tasks, revealing strong efficiency performance trade-offs for both video-only and video language jobs [25]. These findings give significant rationale for adopting SSM-based temporal modeling into deepfake detection systems, particularly for high-resolution, long video inference settings. To our knowledge, the suggested approach is one of the first to integrate SSM-inspired temporal sequence modeling driven by the efficiency concepts of Mamba and VideoMamba directly into a deepfake video forensics pipeline.

### 2.6. Sinkhorn–Knopp Normalization and mHC Layers

The Sinkhorn–Knopp algorithm, introduced in 1967 [26], provides an iterative procedure for transforming any positive matrix into a doubly stochastic matrix one in which all row and column sums equal one. The principle underpinning this approach guarantees that for each *n* × *n* matrix with strictly positive entries, unique diagonal scaling matrices exist such that their product is doubly stochastic. Doubly stochastic matrices possess row and column sums that total one, facilitating equitable dissemination of information among channels. This study empirically assesses the practical significance of this attribute by ablation and efficiency evaluation.

The notion of manifold constrained hyper connections (mHC) was established in the context of big language model training to stabilize residual stream dynamics [27]. The mHC layer uses Sinkhorn–Knopp normalization to a learnable weight matrix at each layer, ensuring that feature information is disseminated across channels in a balanced, norm-preserving way. A follow up suggestion, mHC lite (2026), substitutes iterative Sinkhorn approximation with an exact Birkhoff von Neumann architecture, minimizing convergence hazards and enhancing throughput [28]. The entropy of the doubly stochastic matrix provides a natural measure of information redistribution diversity: high entropy indicates uniform mixing across all channels, whereas low entropy shows concentration of feature flow. We repurpose this entropy metric as a diagnostic signal for adaptive depth expansion in the proposed framework.

### 2.7. FastICA and Feature Initialization

Independent Component Analysis (ICA) and its efficient variation FastICA deconstruct a multivariate signal into components that are as statistically independent as feasible, maximizing higher order non-Gaussianity rather than merely decorrelating (as PCA does). FastICA achieves cubic or quadratic convergence significantly quicker than gradient descent-based ICA without requiring a step size parameter [29]. It finds independent components for nearly every non-Gaussian distribution using a user-selected nonlinearity and can estimate components independently to save computing effort.

ICA has been employed in fMRI brain network identification, EEG artifact reduction, microarray feature extraction, and representation learning [30]. SSM FastICANet enhances deep feature representations by employing FastICA to extract independent components, which are then rigorously filtered via statistical metrics (kurtosis, variance, entropy) to populate, rather than randomly initialize, layer matrices within a State-Space Model framework. This hybrid approach, described in [31], features adaptive, dynamic block growth for improved convergence and, according to the author’s findings, superior interpretability compared to standard architectures.

### 2.8. Adaptive and Progressive Network Growth

The core difficulty in AI development is static overengineering, where deep neural networks are predesigned for “worst case” complexity regardless of the actual objective. This “one size fits all” approach compels small datasets to run through enormous architectures, leading to severe computational waste and models too big for edge devices. To solve this, many techniques have emerged: Optimally Deep Networks (ODNs) [32] dynamically develop a model during training to fit dataset complexity, while Neural Architecture Search (NAS) [33] automates the design process beforehand to identify optimal structures. Alternatively, pruning and quantization optimize models after training by eliminating superfluous components, while early exit methods allow simpler data samples to escape deeper layers during inference [34]. Together, these strategies shift AI toward a more adaptive and resource efficient paradigm. LipGrow (ICML 2020) suggested adaptive residual network training guided by Lipschitz constants, with theoretically valid growth scheduling based on depth-specific performance metrics [35]. Deep Progressive Training (Meta 2025) explores depth expansion from a zero/one layer initialization, presenting theoretical convergence guarantees and insights on initialization schemes, hyperparameter transfer, and growth timing [36]. P DARTS in neural architecture search employs progressive depth expansion with narrowing search spaces to find efficient structures [37]. The proposed framework extends these principles to the deepfake detection domain using entropy-guided growth requirements and ICA-based initialization that are specific to the feature mixing dynamics of the detection challenge.

### 2.9. Synthesis and Positioning of AEGIS-FD

Table 1 illustrates the primary detector categories across four critical dimensions pertinent to video deepfake analysis. Frame-level CNNs are economical however oblivious to temporal signals and exhibit subpar generalization (average AUC = 56.9% on Celeb-DF [8]). Frequency-artifact techniques diminish in efficacy when subjected to compression and contemporary neural rendering. CNN–LSTM hybrids and 3D CNNs use temporal modeling; nonetheless, they depend on spatial features that are independent of frames or experience exponential parameter expansion. Video transformers attain high precision at a quadratic expense for sequence length and generally require extensive pretraining. Models based on SSM provide linear-time temporal representation; nonetheless, similar to all previous families, they establish their depth in advance. No current familial structure adjusts its capabilities to the information during the training process.

The primary methodological innovation of AEGIS-FD lies not in any individual element but in the cohesive control loop established by the components: the Sinkhorn-normalized mHC layer quantifies feature mixing via its entropy; this entropy, in conjunction with validation-AUC fluctuations, influences the decision to augment capacity; and FastICA transforms existing feature statistics into a disentangled initialization for each subsequent layer, thereby stabilizing the entropy signal that directs further expansion. Progressive growing methods (LipGrow [35], Deep Progressive Training [36]) provide growth schedules but no information-theoretic trigger; mHC [27] provides balanced mixing but is static; FastICA [29] is an initialization tool without a growth role. AEGIS-FD is, as far as we are aware, the inaugural framework to integrate these three techniques into a unified self-regulating adaptive-depth process, and the first to substantiate adaptive depth in comparison to matching fixed-depth capacity in deepfake forensics.

## 3. Methodology

To accomplish the objective of robust deepfake face detection from videos, this section presents the methodological framework adopted in this study. We begin by formally defining the problem, followed by a description of the dataset and the preprocessing steps. We then detail the method to design the proposed Adaptive Entropy-Guided ICA SSM Forgery Detector (AEGIS-FD) and its training strategy.

### 3.1. Problem Formulation

We have a video clip $X\in R^{H\times W\times 3\times T}$, where H×W is the spatial dimension of each RGB frame and T is the number offrames (temporal dimension). The question is whether the face in the clip is real (authentic) or deepfake (fake).

Let Y={0, 1}, where 0 stands for real and 1 represents deepfake. The problem is to design a deepfake face detector as a mapping $f_{\theta }:R^{H\times W\times 3\times T}\to Y$ such that $f_{\theta }(X)=y$, where θ are the learnable parameters. The mapping $f_{\theta }$ takes a video clip $X\in R^{H\times W\times 3\times T}$ and predicts its label y∈Y.

The mapping $f_{\theta }$ is typically designed using either traditional machine learning or deep learning. In either case, the architecture of $f_{\theta }$ is fixed and is not adapted to the complexity of the problem. We propose a method to adaptively learn the architecture of $f_{\theta }$ from the data so that it is adapted to the complexity of the data for encoding robust representations.

### 3.2. Dataset and Preprocessing

For the development of $f_{\theta }$, we employ the Celeb-DF v2 benchmark, which consists of three sets: Celeb-real (590 videos) and YouTube-real (300 videos), both assigned label 0 (authentic), and Celeb-synthesis (5639 videos) assigned label 1 (fake). The dataset is divided at video level into training (70%), validation (15%), and test (15%) subsets by stratified random sampling with a seed of 42. The positive class weight $w_{p}$ is calculated solely from the training subset. The ratio of video-level imbalance remains uniform throughout all three divisions (about 6.3:1, fake to real), validating that stratified sampling maintained class equilibrium at the video level. Due to the variability in clip counts based on the duration of each video, the quantity of clips produced every split does not proportionately correspond to the number of source videos; however, this does not influence the fundamental class ratio inside each split. To ensure reproducibility, the assignment of each video to the train, validation, and test sets is documented in a CSV file and finalized before any feature extraction or training begins. Summary statistics of the dataset and split are provided in Table 2. A video deepfake face detector must decide how to sample temporal information from variable length clips. Dense sampling (every frame) is computationally prohibitive; local window sampling misses global temporal patterns; uniform sparse sampling preserves temporal coverage at fixed cost. We use uniform sparse sampling with T = 16 frames. The parameter T can be augmented (for example, to 24 or 32 for extended films) at a linear SSM expense and a quadratic mixing expense. Section 4.10 presents a sensitivity analysis across T ∈ {8, 16, 24}, demonstrating that T = 16 maintains AUC variation within 0.009 points of the extremes while adhering to GPU memory limitations.

For example, if N_f = 100 and T = 16, then i_t = 0, 7, 13, 20, …, 99, uniformly selecting T frames across the full temporal extent of the video. Each selected frame is: (1) converted from BGR to RGB; (2) cropped to the face region using Haar Cascade detection with a 20% margin; (3) resized to 112 × 112 pixels; (4) converted to a PIL Image; (5) augmented (training only) with random horizontal flip and color jitter; (6) normalized with ImageNet statistics μ = [0.485, 0.456, 0.406], σ = [0.229, 0.224, 0.225].

The T processed frames are stacked into a video tensor X ∈ R(T × 3 × H × W), following the standard PyTorch 2.13.0 convention for video batches where the temporal dimension precedes the channel dimension, consistent with 3D convolutional input expectations. If less than T frames are decoded, the most recent valid frame is duplicated to occupy the remaining locations and preserve a consistent tensor structure. Replication is favored over zero-padding as it maintains the statistical distribution of actual frame content, hence preventing the introduction of bogus black frames that may generate spurious temporal distortions and skew the model’s focus towards identifying padding instead of authentic deepfake signals. If no frames are decoded, a zero tensor is returned. These fallback operations ensure robustness to corrupted or very short clips without discarding samples.

### 3.3. Design of the Proposed Model

**Figure 1:**
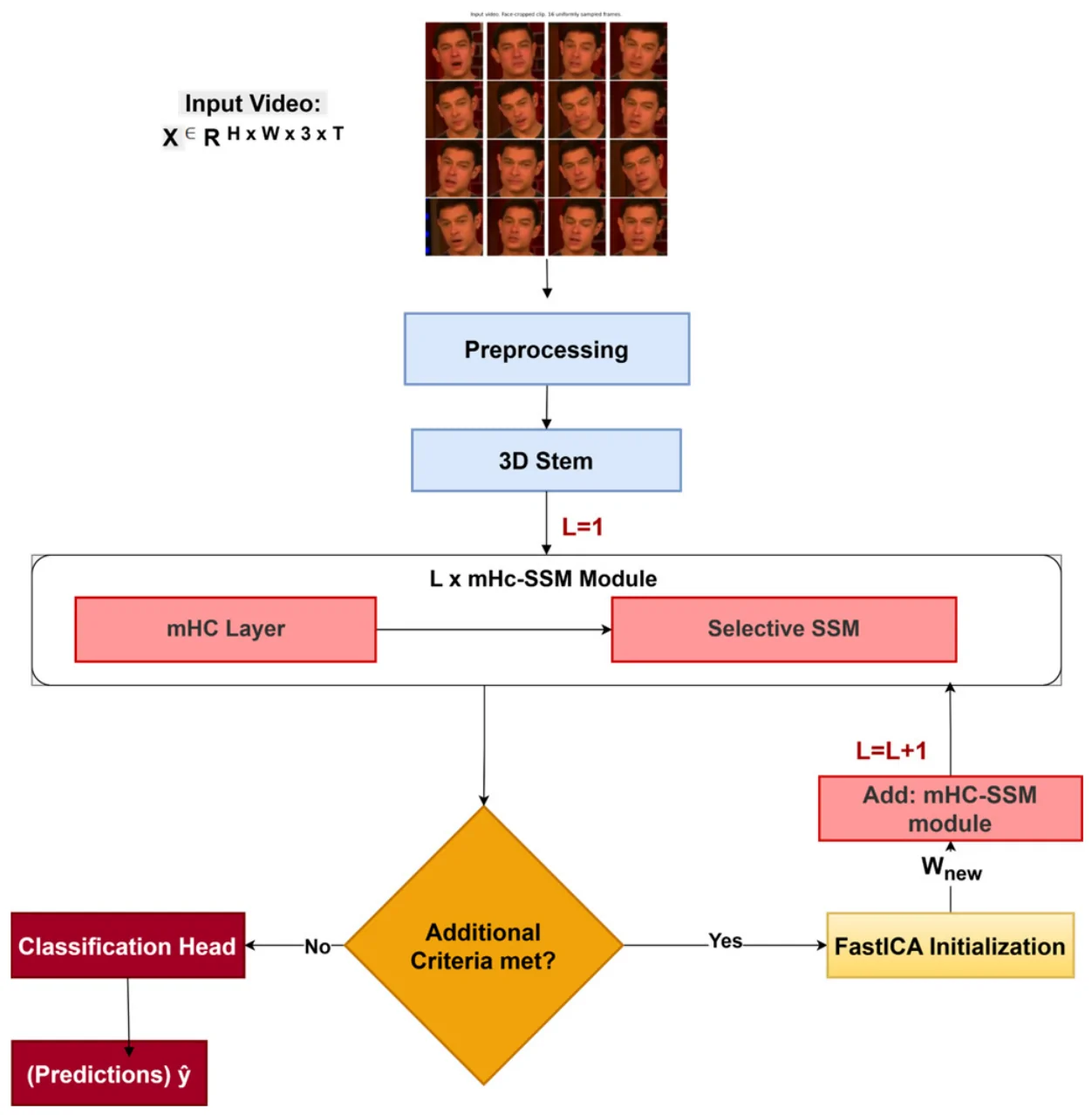
Overview of the Adaptive Entropy-Guided ICA SSM Forgery Detector (AEGIS-FD) architecture.

This presents the detail of the method to design Adaptive Entropy-Guided ICA SSM Forgery Detector (AEGIS-FD) for mapping $f_{\theta }$. The method involves multiple steps, as shown in the diagram in Figure 1 and Algorithm 1. The main components are 3D stem, mHC layer, selective SSM, FastICA initialization and entropy-guided growth, and classification head. The following subsections detail these components.


**Algorithm 1.** Adaptive Entropy-Guided ICA SSM Forgery Detector (AEGIS-FD)**Input:** Deepfake face video dataset D(Train/Val/Test splits); base model M**Output:** Final model $M_{final}$, threshold $\tau^{*}$, test metrics*In experiments,* D *is instantiated as Celeb-DF v2.*
**Processing:**
1: Initialize M with a 3D stem, one mHC–SSM stage (L=1), and a sigmoid head2: Set $E_{max}=50$, patience P=10, warmup $e_{warm}=2$, max depth $L_{max}=8$3: **while** epoch $\leq E_{max}$ **and** early-stop counter <P **do**4:  Train M on $D_{train}$ for one epoch5:  $\left(val\_AUC,H\left(e\right)\right)\leftarrow EVALUATE\left(M,D_{val}\right)$6:  Update early-stop counter; save checkpoint if val_AUC improves7:  **if** epoch $\geq e_{warm}$ **then**8:   $\Delta AUC\leftarrow \frac{1}{3}\sum_{k=0}^{2}\Delta AUC\left(epoch-k\right)$9:   $\Delta H_{rel}\leftarrow \frac{|H\left(e\right)-H\left(e-1\right)|}{H\left(e-1\right)}$10:   **if** 0.005≤ΔAUC≤0.05 **and** $\Delta H_{rel}\geq \tau_{entropy}$ **and** $L<L_{max}$ **then**11:     $Z^{\prime \prime }\leftarrow EXTRACTTOPLAYERFEATURES\left(M,D_{train},N_{s}=2000\right)$12:     $W_{init}\leftarrow FASTICA\left(Z^{\prime \prime }\right)$13:     Add new mHC–SSM stage initialized with $W_{init}$14:     L←L+115:     Re-initialize optimizer and scheduler16:   **end if**17:  **end if**18: **end while**19: $M_{final}\leftarrow$ best checkpoint (max val_AUC)
**Threshold optimization and testing:**
20: $Val\_Probs\leftarrow PREDICT\left(M_{final},D_{val}\right)$21: $\tau^{*}\leftarrow \arg\;{max}_{\tau \in \left[0.10,0.90\right]}F1SCORE\left(Val\_Probs,D_{val},labels\;\tau \right)$22: $TestMetrics\leftarrow EVALUATEWITHTHRESHOLD\left(M_{final},D_{test},\tau^{*}\right)$23: **return** $\left(M_{final},\tau^{*},TestMetrics\right)$


#### 3.3.1. 3D Stem

2D CNNs process frames independently and cannot model joint spatial temporal patterns within a single forward pass. 3D CNNs apply volumetric kernels that convolve jointly over the spatial and temporal dimensions of a video clip, enabling the detection of motion artifacts, temporal flickering, and inter-frame boundary inconsistencies introduced during synthesis.

The video clip $X\in R^{3\times T\times H\times W}$ is passed through the *3D stem*:

Stem(X)=AdaptPool(ReLU(BN(Conv3D(X))))

where *Conv3D* contains 64 kernel with (3, 5, 5) size, stride (1, 2, 2), and padding (1, 2, 2), BN denotes batch normalization, and *AdaptPool* applies spatial adaptive average pooling that transforms the spatial size to 8 × 8 while preserving the temporal dimension *T*. The output has shape $R^{64\times T\times 8\times 8}$, which is reshaped to $R^{T\times 4096}$ (where 4096 = 64 × 8 × 8) and projected to $Z\in R^{T\times d}$ (128) via a linear layer. Precisely, it is described as:

Z=Reshape(Stem(X),T,64×8×8)W+b

where $W\in R^{d\times 4096}$ is the learnable projection weight matrix and $b\in R^{d}$ is the bias vector.

The 3D stem generates a basic temporal feature sequence $Z\in R^{T\times d}$, where each row $Z_{t}$ is a d-dimensional spatiotemporal embedding of frame t, 1≤t≤T. The sequence Z is used as input mHC-SSM component consisting of L stacked modules of mHC-SSM, each including a mHC channel-mixing layer succeeded by a selected SSM temporal block, as detailed in Section 3.3.2 and Section 3.3.3, respectively.

#### 3.3.2. mHC: Manifold Constrained Hyper Coupling Layer

In traditional residual networks, interactions among feature channels occur implicitly through convolutional filters. The mHC layer renders this interaction explicit and manageable by learning a per-layer channel mixing matrix $W\in R^{T\times T}$, where T=16 represents the number of sampled frames; coupling occurs across the temporal dimension rather than the feature dimensions. Enforcing doubly stochasticity by Sinkhorn–Knopp normalization (see Algorithm 2) guarantees that the mixing matrix M possesses a spectral norm of precisely 1. This establishes a limit on the operator norm, ‖MZ‖ ≤ ‖M‖_2_‖Z‖ = ‖Z‖, ensuring the mHC layer cannot amplify feature magnitudes during mixing, which contributes to stable gradient propagation across the network’s growing depth. In our environment, this facilitates equitable information transfer among frames, and the efficacy of the mHC layer is evaluated empirically by ablation and efficiency outcomes. From an information-theoretic viewpoint, the entropy of a doubly stochastic matrix reaches its peak with uniform distribution across all channel pairs and diminishes steadily as the matrix becomes concentrated on a limited set of predominant mixing pathways. A plateauing entropy signifies that the channel-mixing architecture of the current layer has reached a stable, non-uniform arrangement that mirrors the qualities it has learned to emphasize.

Formally, each mHC layer maintains a learnable weight matrix $W\in R^{T\times T}$. The Sinkhorn normalization procedure is defined as:

$$P^{\left(0\right)}\;=\;|W|\;+\;\varepsilon ,$$

where ∣W∣ denotes the matrix of element-wise absolute values, i.e., ${|W|}_{ij}\;=\;|w_{ij}|$, and ε>0 is a small constant added to ensure strict positivity required by the Sinkhorn–Knopp algorithm.

The Sinkhorn iterations apply alternating column and row normalization operators defined as:


$$T_{c}{\left(P\right)}_{ij}=\frac{P_{ij}}{\sum_{k=1}^{T}P_{kj}},T_{r}{\left(P\right)}_{ij}=\frac{P_{ij}}{\sum_{k=1}^{T}P_{ik}}$$

At each iteration $P^{\left(k\right)}=T_{r}\;\left(T_{c}\left(P^{\left(1\right)}\right)\right)$ and ${M=P}^{\left(k\right)}$ after K=5 iterations.

After converting W to the doubly stochastic matrix M via Sinkhorn–Knopp normalization, the input temporal feature sequence $Z\in R^{T\times d}$, where each of the T=16 rows is a d-dimensional feature vector of one frame, is mixed across frames as:


$$Z^{\prime }=M\cdot Z$$

The (j)-th entry $M_{ij}$ specifies the weight given to frame j when computing the new mixed representation of frame i. Because M is doubly stochastic, $Z_{i}^{\prime }$ is a convex combination of all T frame representations (row sums = 1), and every frame contributes equally in total across all output frames (column sums = 1). The output $Z^{\prime }\in R^{T\times d}$ is then passed to the selective SSM block.

The mixing entropy is defined as


$$H\left(M\right)=-\sum_{i,j}M_{ij}\;log\;\left(M_{ij}\right).$$

This measure the consistency of the mixing matrix in information dissemination: the peak, achieved by the uniform matrix $M_{ij}=1/T,\;is\;H_{max}=T\cdot logT$, (the maximum log T per row aggregated over T rows; 16·log 16 ≈ 44.36 nats for T = 16), while diminished entropy indicates a constrained flow concentrated in a narrow range of entries.


**Algorithm 2.** Sinkhorn–Knopp Normalization for mHC Layer**Input:** Non-negative affinity matrix $K\in R^{d\times d}$, number of iterations $N_{\mathrm{iter}}$, stability constant ϵ**Output:** Doubly stochastic normalized coupling matrix $P\in R^{d\times d}$
**Processing:**
1: Initialize row scaling vector $u^{\left(0\right)}\leftarrow 1_{d}$(a vector of ones of size d)2: Initialize column scaling vector $v^{\left(0\right)}\leftarrow 1_{d}$3: **for** t=1 **to** $N_{\mathrm{iter}}$ **do**4:  Row Update:5:   $u^{\left(t\right)}\leftarrow 1_{d}⊘\left(Kv^{\left(t-1\right)}+\epsilon \right)$6:  Column Update:7:   $v^{\left(t\right)}\leftarrow 1_{d}⊘\left(K^{⊤}u^{\left(t\right)}+\epsilon \right)$8: **end for**9: Construct the final normalized matrix:10: $P\leftarrow diag\left(u^{\left(N_{\mathrm{iter}}\right)}\right)\times K\times diag\left(v^{\left(N_{\mathrm{iter}}\right)}\right)$11: **return** P**Note:** ⊘ denotes element-wise division.


#### 3.3.3. Selective SSM Block

Following the channel mixing via the mHC layer, the temporal sequence $Z^{\prime }\in R^{T\times d}$ necessitates a module that encodes sequential dependencies across the T = 16 frame locations. We utilize a selective state space block that implements three essential mechanisms of Mamba architecture: (1) input-dependent state parameters calculated for each token, facilitating selective propagation and retention; (2) a causal depthwise convolution branch that offers local temporal context preceding the SSM step explained in Algorithm 3 below; and (3) a content-dependent output gate that regulates the SSM pathway utilizing the original input. The block is executed in pure PyTorch as a sequential recurrence across T steps. For T = 16, this recurrence is computationally insignificant, and its mathematical characteristics align with those of a parallel scan implementation; no hardware-specific kernels are necessary.

First, the input $Z^{\prime }\in R^{T\times d}$ is projected into two streams simultaneously via a single linear layer:

$$[U_{s}∥U_{g}]=Z^{\prime }W_{in}^{⊤}+1\beta_{in},U_{s},U_{g}\in R^{T\times H}$$

where $U_{s}$ is the SSM stream and $U_{g}$ is the gate stream, and H is the inner dimension of the block. Then the SSM stream $U_{s}$ is processed by a causal depthwise convolution with kernel size k=4:


$$V=CausalDepthwiseConv\left(U_{s},k=4\right),V\in R^{T\times H}.$$

This provides each frame position with local temporal context from up to 3 preceding frames before the recurrence step. Then the hidden state $h\in R^{d_{s}}$ is initialized to zero. For each frame t=1,…,T, the input-dependent SSM parameters are computed from $V_{t}$:


$$[a_{t},b_{t},c_{t}]=V_{t}W_{p}^{⊤},a_{t},b_{t},c_{t}\in R^{d_{s}}$$

The state is then updated recurrently:

$$h_{t}=\sigma (a_{t})⊙h_{t-1}+tanh(b_{t})$$

where $\sigma (a_{t})$ acts as a selective forget gate controlling how much of the previous state is retained and $tanh(b_{t})$ is the input update.

The output at frame t is read from the state via:


$$y_{t}^{state}=tanh(c_{t})⊙h_{t}$$

$$y_{t}=W_{o}\;y_{t}^{state},y_{t}\in R^{H}$$

The three parameters $a_{t},b_{t},c_{t}$ are all computed from the current frame input $V_{t}$, making the state transitions fully input-dependent; this is the selectivity mechanism that distinguishes this block from standard fixed-parameter SSMs.

After collecting all frame outputs, the SSM result is gated by the gate stream $U_{g}$:


$$Y_{ssm}=Stack(y_{1},\ldots ,y_{T}),Y_{ssm}\in R^{T\times H}$$

$$Z_{gated}=Y_{ssm}⊙\sigma \left(U_{g}\right).$$

This content-dependent gate regulates how much of the SSM output is passed forward, based on the original input projection.

The gated output is projected back to the d-dimensional space, and a residual connection with LayerNorm stabilizes training:


$$Z_{out}=Z_{gated}\;W_{out}^{⊤},{\;Z}_{out}\in R^{T\times d},$$

$$Z^{\prime \prime }=LayerNorm\left(Z^{\prime }+Dropout(Z_{out},p=0.2)\right).$$

The output $Z^{\prime \prime }\in R^{T\times d}$ is passed to the next mHC-SSM stage, or to the classification head after the final stage. Algorithm 3 describes the detail of a forward pass of selective SSM Block.


**Algorithm 3.** Selective SSM Block**Input:** sequence $Z^{\prime }\in R^{T\times d}$, projection weights $W_{in},\beta_{in},W_{p},W_{o},W_{out}$**Output:** sequence $Z^{\prime \prime }\in R^{T\times d}$
**Input Projection and Stream Split:**
1: $\left[U_{s}∥U_{g}\right]\leftarrow Z^{\prime }W_{in}^{⊤}+1\beta_{in},\;U_{s},U_{g}\in R^{T\times H}$
**Causal Depthwise Convolution:**
2: $V\leftarrow CausalDepthwiseConv\left(U_{s},k=4\right),\;V\in R^{T\times H}$
**Initialize State:**
3: $h_{0}\leftarrow 0\in R^{d_{s}}$4: **for** t=1 **to** T **do**
  **Selective parameterization (from **
$V_{t}$
**):**
5:   $\left[a_{t},b_{t},c_{t}\right]\leftarrow V_{t}W_{p}^{⊤},\;a_{t},b_{t},c_{t}\in R^{d_{s}}$
  **Gated state update:**
6:   $h_{t}\leftarrow \sigma \left(a_{t}\right)⊙h_{t-1}+tanh\left(b_{t}\right)$
  **Readout:**
7:   $y_{t}^{state}\leftarrow tanh\left(c_{t}\right)⊙h_{t}$8:   $y_{t}\leftarrow W_{o}y_{t}^{state},\;y_{t}\in R^{H}$9: **end for**
**Collect Outputs and Apply Content-Dependent Gate:**
10: $Y_{ssm}\leftarrow Stack\left(y_{1},\ldots ,y_{T}\right),\;Y_{ssm}\in R^{T\times H}$11: $Z_{gated}\leftarrow Y_{ssm}⊙\sigma \left(U_{g}\right)$
**Output Projection, Residual, and Normalization:**
12: $Z_{out}\leftarrow Z_{gated}W_{out}^{⊤},\;Z_{out}\in R^{T\times d}$13: $Z^{\prime \prime }\leftarrow LayerNorm\left(Z^{\prime }+Dropout\left(Z_{out},p=0.2\right)\right)$14: **return** $Z^{\prime \prime }$**Note:** $\sigma \left(a_{t}\right)$: selective forget gate; $tanh\left(b_{t}\right)$: input update.


#### 3.3.4. Adaptive Architecture Growth

Upon the completion of a mHC-SSM module, which yields an output $Z^{\prime \prime }\in R^{T\times d}$, the framework determines whether to incorporate and stack another mHC-SSM module or stop. A static shallow architecture may be insufficient to capture the intricate temporal artifact patterns present in deepfake face videos, whereas a static deep design may overfit to the limited authentic video subset—containing only 890 real videos compared to 5639 fake ones. Progressive growth enables the model to commence with simplicity, so minimizing the risk of early overfitting, and to augment its capability only when validation data substantiates the necessity.

The suggested growth criteria are data-adaptive: they initiate growth solely when the model continues to learn significantly (positive AUC increase) and the feature mixing remains dynamic (non-stationary entropy), hence averting unnecessary layer additions once training has settled.

The application of mixing entropy as an indicator of growth is grounded on information theory. For a doubly stochastic matrix M, H(M) reaches its maximum with the uniform matrix and its minimum with permutation matrices; thus, it measures the uniformity of information distribution across frames. Two characteristics render it appropriate as a capacity-utilization metric. Initially, according to the Birkhoff–von Neumann theorem, any doubly stochastic matrix can be expressed as a convex combination of permutation matrices, so a converged low-entropy M signifies that the layer has transitioned into a near-permutation (routing) state where additional mixing diversity is depleted. Secondly, while Sinkhorn normalization stabilizes the row and column marginals, alterations in H(M) signify authentic reconfiguration of the mixing pattern instead of mere scale drift; hence, |ΔH| delineates the current representational transformation. Growth is initiated specifically when validation improvements have leveled off while mixing continues to undergo reorganization—the hallmark of a layer at capacity yet with unstable features. Section Entropy Dynamics as an Indicator of Growth substantiates this signal with empirical evidence.

The network is initialized at depth L=1. Growth to depth L+1 at epoch e is triggered only when all three of the following criteria are satisfied simultaneously:

Criterion A—AUC Plateau:

$$\mathrm{Let}\;\Delta_{AUC}^{\left(e\right)}={AUC}^{\left(e\right)}-{AUC}^{\left(e-1\right)}.$$

Growth is triggered when

$$0.005|\;\leq \;|\;\frac{1}{3}\sum_{k=0}^{D}\Delta_{AUC}^{\left(k\right)}|\;\;\leq \;|\;0.05,\;$$

*where D* = 2 *defines the smoothing window* (3 − *epoch moving average*).

This detects a learning plateau—the model has stagnated without diverging. Growth is inhibited when the model is either actively improving (mean gain >0.05) or destabilizing (mean gain <0).

Criterion B—Entropy Dynamism:

$$\mathrm{Let}\;H^{\left(e\right)}=-\sum_{i,j}M_{ij}^{\left(e\right)}logM_{ij}^{\left(e\right)}$$

be the mixing entropy of the last mHC layer at epoch e.

The relative change must satisfy:


$$\frac{|H^{\left(e\right)}-H^{\left(e-1\right)}|}{H^{\left(e-1\right)}}|\;\geq |\;0.001$$

This guarantees that the mixing pattern continues to develop; a static entropy signifies that the present layer has stabilized, thereby inhibiting growth until entropy begins to change actively, alongside Criterion A’s plateau identification.

Criterion C—Depth Ceiling:

$$L|\;<|\;L_{max}=8.$$

Additionally, a warmup period of $e_{warm}=2$ epochs must elapse before any growth is considered, ensuring the initial layer has received adequate gradient signals to yield significant entropy and AUC measures.

Criterion D—validation-gated retention

Meeting Criteria A–C initiates a candidate expansion from depth L to L + 1, commenced through FastICA as outlined in Section 3.3.5. This candidate layer is not preserved without conditions: its approval depends on future validation outcomes. If the validation AUC at depth L + 1 does not surpass the highest validation AUC noted at depth L within a tolerance of 3 epochs, the expansion is denied, the additional layer is eliminated, and the network returns to depth L, which is subsequently fixed as the final architecture for the duration of training. This retention verification inhibits the network from dedicating extra capacity that fails to provide a true generalization advantage, differentiating the suggested mechanism from merely monotonic growth patterns that unconditionally increase depth upon the fulfillment of a trigger condition.

#### 3.3.5. FastICA Initialization

Upon the fulfillment of all three criteria, a new mHC-SSM module is added to the stack. The random initialization of the new mHC weight matrix $W_{new}$ results in an essentially uniform doubly stochastic matrix M at maximum entropy, causing the new layer to function initially as an identity pass-through and prolonging convergence since the network must learn non-trivial mixing from the beginning.

FastICA extracts statistically independent components from a multivariate signal by maximizing non-Gaussianity, attaining quadratic convergence without necessitating a step size. Upon the initiation of growth, the existing intermediate feature matrix $Z^{\prime \prime }\in R^{N_{s}\times d}$ (capped at $N_{s}=2000$ samples) is passed to FastICA. The current intermediate feature matrix $Z^{\prime \prime }\in R^{N_{s}\times d}$ is first transposed and reshaped to $R^{N_{s}\times T}$ by mean-pooling the d feature dimensions into T components before being passed to FastICA. The returned component matrix $A_{ICA}\in R^{T\times T}$ then initializes the new mHC weight matrix: $W_{new}\leftarrow A_{ICA}$.

This indicates that the next layer initiates from a configuration that already allocates frame representations along statistically independent axes, providing a more informative foundation than randomness. In the case of deepfake face detection, where genuine and fabricated temporal features are anticipated to have divergent statistical patterns, ICA initialization facilitates the earlier disentanglement of these distributions in the new layer. The FastICA function is restricted to 300 iterations with a convergence tolerance of 0.005. Xavier uniform initialization acts as a contingency when FastICA fails to converge, guaranteeing robustness without hindering training.

To address the constraints of stochastic initialization, which frequently results in redundant feature acquisition, we implement a disentangled initialization technique for each new mHC-SSM stage. We employ Fast Independent Component Analysis (FastICA) to initialize the new module’s weights $W_{init}$ according to the statistics of the activations from the preceding layers. This method transforms intermediate features into a collection of statistically independent components, guaranteeing that the newly incorporated layers are promptly equipped to detect unique forgery artifacts—such as unnatural warping or texture inconsistencies—rather than adopting the feature bias of the existing module. The sequential approach for this initialization is outlined in the Algorithm.

Following the addition of a new module, the optimizer and learning rate scheduler are reinitialized to reset momentum states, ensuring that the new layer parameters receive proper gradient scaling from the outset. The pseudocode for the growth procedure is presented in Algorithm 4.


**Algorithm 4.** FastICA Initialization for mHC-SSM Module**Input:** Extracted top-layer feature matrix $Z^{\prime \prime }\in R^{N_{s}\times d}$, convergence tolerance ϵ**Output:** Initialized weight matrix $W_{\mathrm{init}}$
**Processing:**
1: **Centering:** Compute the mean of $Z^{\prime \prime }$ and subtract it to yield zero-mean data X2: **Whitening:** Apply eigenvalue decomposition to the covariance matrix of X3: Transform X into whitened data $\tilde{X}$ such that its covariance is the identity matrix4: Initialize weight matrix $W_{\mathrm{init}}$ randomly (e.g., using Xavier initialization)5: Set error ←∞6: **while** error >ϵ **do**7:   $W_{\mathrm{old}}\leftarrow W_{\mathrm{init}}$8:   $W_{\mathrm{init}}\leftarrow E\left[\tilde{X}g\left(W_{\mathrm{init}}^{T}\tilde{X}\right)\right]-E\left[g^{\prime }\left(W_{\mathrm{init}}^{T}\tilde{X}\right)\right]W_{\mathrm{init}}$9:   $W_{\mathrm{init}}\leftarrow \frac{W_{\mathrm{init}}}{∥W_{\mathrm{init}}∥}$10:   error $\leftarrow 1-|W_{\mathrm{init}}^{T}W_{\mathrm{old}}|$11: **end while**12: **return** $W_{\mathrm{init}}$
**Notes:**

g is a non-linear contrast function

Line 9 performs orthogonalization and normalization

Line 10 checks for convergence



#### 3.3.6. Classification Head

The stacked L mHC-SSM modules produce the final output sequence $Z^{\prime \prime }\in R^{T\times d}$, temporal evidence is aggregated across all T=16 frame positions via mean pooling:


$$z^{-}=\frac{1}{T}\sum_{t=1}^{T}z_{t}^{\prime \prime },z^{-}\in R^{d}.$$

Mean pooling was favored over max pooling due to the distribution of deepfake artifacts across numerous frames, rather than their concentration in a single frame, rendering the average a more resilient summary of temporal evidence. Attention pooling was dismissed because of the introduction of learnable characteristics at the final detection stage, thereby heightening the risk of overfitting in light of the limited availability of real video data. The aggregated representation $z^{-}$ is sent through a dropout layer and a linear classifier to generate the chance of being fake:

$$\hat{y}=\sigma \;\left(W_{c}\;Dropout(z^{-},p=0.3)+b_{c}\right),\hat{y}\in [0,\;1]$$

where $W_{c}\in R^{1\times d}$, $b_{c}\in R$, and σ is the sigmoid function. Dropout with rate 0.3 at this stage provides the final regularization before the binary decision, reducing sensitivity to specific feature activations in the last layer of the stack.

#### 3.3.7. Training Strategy

The Celeb-DF v2 training dataset has roughly 6.33 fake videos for each authentic video. The use of standard unweighted binary cross-entropy to imbalanced data results in the model prioritizing the accurate classification of the majority class (fake) while inadequately learning the minority class (real), enhancing recall at the cost of precision. To overcome this issue, the weighted binary cross-entropy loss is used for training:

$$L=-\frac{1}{N}\sum_{i=1}^{N}\left[w_{p}\;y_{i}log{\hat{y}}_{i}+(1-y_{i})log(1-{\hat{y}}_{i})\right]$$

where the positive class weight is calculated directly from the training subset:


$$w_{p}=\frac{N_{real}}{N_{fake}}=\frac{623}{3947}\approx 0.158$$

Given that $y_{i}$ = 1 signifies counterfeit videos, utilizing $w_{p}$ for the positive-class term as $w_{p}$ ≈ 0.158 diminishes the relative gradient impact of the predominant (fake) class, thereby amplifying the relative significance of the minority (real) class throughout the optimization process and remedying its insufficient representation in the training dataset.

The specifications presented in Table 3 are enhanced through the application of AdamW, with a learning rate of 10^−4^, a weight decay of 10^−4^, and gradient norm clipping established at 1.0. The implementation of mixed precision training through the torch autocast function lessens GPU memory requirements and enhances training speed in the presence of CUDA. The entirety of the tests was carried out in PyTorch 2.13.0, utilizing CUDA 13.0 and cuDNN 9.2.0, and conducted on a single NVIDIA Quadro RTX 6000 GPU. The ReduceLROnPlateau function decreases the learning rate by a factor of 0.5 when validation AUC fails to improve for three consecutive epochs, facilitating precise adjustment in subsequent training phases. Early stopping halts training if the validation AUC fails to improve for 10 successive epochs, hence retaining the optimal model checkpoint.

The classifier output $\hat{y}\in [0,\;1]$ is converted to a binary label using a threshold τ:


$$\hat{l}=1\left[\hat{y}\geq \tau \right].$$

The conventional sigmoid threshold of 0.5 is infrequently optimal in situations of class imbalance. The best threshold is determined on the validation set as follows:

$$\tau^{*}=arg\;{max}_{\tau \in \{0.10,\;0.11,\;\ldots ,\;0.90\}}F_{1}(\tau )$$

where $F_{1}(\tau )$ denotes the $F_{1}$ score calculated at threshold τ. This equilibrates precision (preventing erroneous allegations of genuine information) and recall (identifying fraudulent content). The selected $\tau^{*}$ is thereafter utilized without alteration during testing, guaranteeing that no information from the test set affects threshold determination.

A universal random seed of 42 is utilized for operations, as well as for the dataset partition generator. The comprehensive hyperparameter configuration is given in Table 3.

### 3.4. Program for Experimental Research

To evaluate the influence of each component and to tackle the central question of Section 1—whether entropy-driven growth improves generalization beyond a comparable fixed-depth capacity—the analysis follows a specified sequence of ten experiments, E1–E10, all employing the identical data split, preprocessing framework, and hyperparameters as detailed in Table 3 unless stated otherwise. Section 4.12, located at the conclusion of Section 4, encapsulates the aims, setups, and reporting sites of every experiment.

## 4. Results and Discussion

This section presents and analyzes the findings of the suggested approach, structured in accordance with the experimental framework outlined in Section 3.4. We commence with an exposition on the evolution and training of AEGIS-FD (Section 4.1), succeeded by a quantitative performance evaluation that includes component-wise ablation (E1) and a growth-versus-matched-fixed-depth analysis (E2), both elaborated in Table 4, which specifies component-specific performance on Celeb-DF v2 (Section 4.2). We subsequently examine the unique influence of FastICA initialization (Section 4.3) and corroborate the entropy-guided growth signal via an entropy-trajectory analysis (E6, Section Entropy Dynamics as an Indicator of Growth), succeeded by an error and confusion-matrix evaluation (E6, Section 4.4). The establishment of multi-seed statistical robustness (E3) is detailed in Section 4.5, while the evaluation of zero-shot cross-dataset transferability (E4) is conducted in Section 4.6. Section 4.7 assesses the computational expense associated with adaptive growth (E5). Ultimately, Section 4.8 examines the halting criterion and the trade-off between complexity and accuracy; Section 4.9 evaluates options for addressing imbalance (E7); Section 4.10 presents sensitivity analyzes (E9); Section 4.11 describes the methodology within the framework of prior studies; early-exit inference (E10) was not subjected to empirical assessment in this research and is mentioned as a potential avenue for subsequent investigation (Section 5.2); and Section 4.12 contextualizes the method in relation to previous research.

### 4.1. Development and Training of AEGIS-FD

AEGIS-FD underwent training for a maximum of 50 epochs, utilizing early stopping that monitored validation AUC to cease training when no additional enhancement was seen within a specified patience window, as detailed in Section 3.3.6. The network’s depth incrementally increased throughout training, influenced by the entropy-driven growth standard: expansion occurrences were initiated as the network attained an ultimate depth of L = 6. No other growth events transpired after this juncture, and the network sustained a depth of 6 for the duration of training. The ideal checkpoint was noted at epoch 31, where the model achieved its peak validation AUC of 0.9607 while functioning in this depth-6 setup. Early stopping subsequently terminated training when the validation AUC did not enhance during the subsequent patience interval, indicating that depth-6 constituted a stable, well-converged configuration rather than a transient stage in an evolving architecture. This checkpoint was preserved as the ultimate deployed model and assessed on the reserved test set, producing a test AUC of 0.9600, in alignment with the validation-time outcome.

### 4.2. Quantitative Performance Analysis

Table 4 showcases a component-specific ablation analysis employing Celeb-DF v2, which now incorporates the training expense per configuration measured in GPU-hours on a solitary Quadro RTX 6000. Incorporating the mHC layer into the base SSM (A1 → A2) results in an improvement of +3.00 AUC points (0.84 → 0.87) and +3.05 accuracy points (84.49% → 87.54%), demonstrating that Sinkhorn-normalized doubly stochastic channel mixing offers significant advantages via norm-preserving gradient stabilization and entropy-driven growth signaling, with a slightly reduced training expense (0.21 → 0.18 GPU-hours), as the additional mHC layer does not extend the sequential recurrence length.

Facilitating adaptive growth via Xavier initialization (A2 → A6) enhances the AUC to 0.89 (+2.00 points), although the model reaches a maximum depth of L = 8 at almost twice the training expense (0.18 → 0.39 GPU-hours), suggesting that random initialization inadequately directs the growth cessation criterion and compels the network to utilize considerable extra computation without a corresponding increase in accuracy.

Substituting Xavier with FastICA initialization (A6 → A7, complete AEGIS-FD) yields the most substantial improvement: +7.00 AUC points (0.89 → 0.96) and +9.20 accuracy points (87.54% → 96.74%), while concurrently decreasing training expenses by 61.5% (0.39 → 0.15 GPU-hours) and achieving convergence at the ideal depth of L = 6 instead of L = 8. This illustrates that statistically independent weight initialization enhances overall performance, regulates the growth process to avert excessive depth proliferation, and significantly reduces the computational resources necessary to achieve that performance—as FastICA-guided growth concludes sooner, requires fewer post-growth epochs to stabilize each new layer, and eliminates the redundant training expended on Xavier’s ultimately abandoned deeper configurations.

To evaluate if adaptive growth has advantages beyond merely achieving depth 6, we juxtapose AEGIS-FD (A7, adaptive growth, AUC 0.96) with a separate fixed-depth-6 model that employs the same mHC and FastICA components but has growth deactivated from the outset (A5, AUC 0.95). AEGIS-FD exceeds the fixed-depth-6 standard by +0.01 AUC, +2.95 accuracy points (93.73% → 96.68%), and +0.021 F1 points (0.9558 → 0.9767), indicating that the entropy-informed development timeline has representational benefits beyond simply matching network capacity. Notably, AEGIS-FD employs 37.5% less GPU time in comparison to the fixed-depth-6 benchmark (0.24 → 0.15 GPU-hours), as the progressive curriculum enables initial layers to resolve a simpler subproblem prior to the introduction of deeper layers, in contrast to the simultaneous optimization of all six layers from the outset in A5. This indicates that adaptive development serves as a legitimate training approach for enhancing final accuracy, while also being more computationally economical; multi-seed replication (Section 4.5) verifies that this AUC benefit is not due to a singular advantageous initialization. This enhancement in AUC and accuracy aligns with the anticipated advantages of norm-preserving channel mixing, but the ablation focuses on the layer’s overall impact on detection efficacy rather than specifically assessing feature-mixing entropy or spectral norm preservation as causal factors.

The initialization of FastICA produces a dual impact. With a fixed depth, the FastICA-initialized depth-6 model achieves a performance of 0.95, surpassing the Xavier-initialized depth-6 model’s 0.84 by 0.11 as summarized in Table 5. In the context of growth, FastICA functions as a depth regulator enhancing AUC from 0.89 to 0.96; FastICA sacrifices the enhanced precision of deep growth for a significantly more compact deployed model.

**Figure 2:**
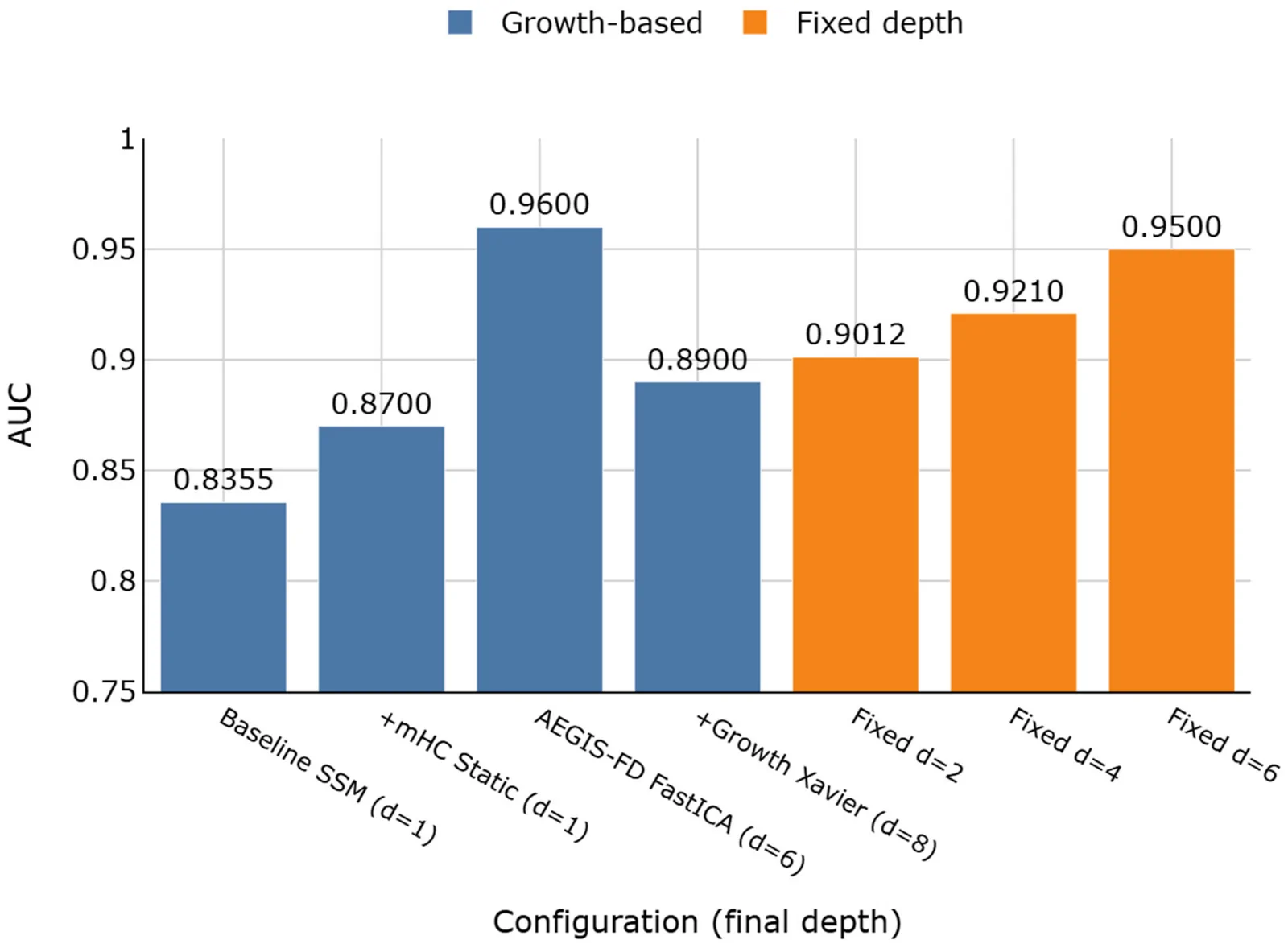
AUC by architectural configuration on Celeb-DF v2, grouped by training strategy. Blue bars denote growth-based configurations (baseline SSM, +mHC, Xavier-growth, and AEGIS-FD/FastICA-growth); orange bars denote independently trained fixed-depth baselines (no growth). Configurations are ordered by final network depth. AEGIS-FD (d = 6, AUC = 0.9600) exceeds the matched fixed-depth-6 baseline (AUC = 0.9500) despite reaching the same final capacity, isolating the contribution of the entropy-guided growth process.

Since the seven configurations in Table 4 represent two experimental conditions, growth-based configurations that arrive at their final depth through the entropy-guided or Xavier-initialized growth process (blue bars) and fixed-depth configurations trained from initialization with growth disabled (orange bars), Figure 2 shows these results as a grouped bar chart rather than a linear plot. As discrete, non-connected bars, these avoid the earlier ambiguity of indicating interpolation between architecturally unrelated configurations (e.g., the Xavier-growth model reaching depth 8 is not the same trajectory as the FastICA-growth model reaching depth 6). At depth 6, AEGIS-FD (0.9600) outperforms the fixed-depth-6 baseline (0.9500) by 0.01 AUC and outperforms the naïve Xavier-growth variation (0.8900) that failed to stop. AEGIS-FD’s performance depends on entropy-guided cessation and FastICA initialization to achieve the correct final depth and the highest AUC.

### 4.3. Impact of FastICA Initialization

A significant discovery of this research is the difference between random and data-informed initialization during the architecture expansion phase. In setup A6 (SSM + mHC + Growth, initialized with Xavier), the model effectively scaled through all growth events but persisted in growing to the maximum permissible depth of L = 8, ultimately achieving a moderate AUC of 0.8900 (Table 4). This suggests that Xavier-initialized new layers, which commence as nearly random, high-entropy transformations, undermine the entropy-dynamism growth criterion (Criterion B, Section 3.3.4) by lacking a dependable stopping signal—the model persists in augmenting capacity without achieving a proportional increase in discriminative efficacy, reaching depth 8 without equaling the performance of the more superficial, FastICA-guided setup.

Conversely, AEGIS-FD (A7) attained its ultimate depth of L = 6 using the validation-gated growth process, which dismissed additional progression to depths 7 and 8 when both configurations demonstrated a decline in validation AUC compared to depth 6. The best validation checkpoint was documented at epoch 31 (validation AUC = 0.9607), indicating that the accuracy improvements noted in Table 4 arise from sustained convergence at this validation-chosen depth-6 configuration, rather than from unrestricted architectural expansion. This suggests that after six layers, the extra capacity surpassed the effective support of the Celeb-DF v2 training set, and early halting accurately recognized this saturation point instead than being misled by non-informative growth signals as shown in A6. By introducing new layers with statistically independent elements sourced from active feature representations, AEGIS-FD circumvented the initial convergence instability commonly associated with progressive growth using random initialization, enabling each new module to promptly differentiate between authentic and fabricated temporal patterns instead of commencing from a non-informative identity-like transformation. This initialization approach results in a significant enhancement in performance: substituting Xavier with FastICA initialization while maintaining the growth mechanism unchanged (A6 → A7) leads to a +7.0 AUC-point increase (0.8900 → 0.9600) and a +9.2 accuracy-point rise (87.54% → 96.74%), concurrently decreasing the final network depth from 8 to 6 layers. In comparison to the single-layer baseline Mamba SSM (A1, AUC = 0.8355), the comprehensive FastICA-guided AEGIS-FD setup achieves an enhancement of +12.45 AUC points overall. These findings indicate that statistically independent weight initialization enhances ultimate performance while also moderating the growth process, thereby averting the excessive depth increase seen with naive random initialization. To verify the growth signal itself, the mixing entropy H(M) of the final mHC layer and validation AUC were tracked jointly across all six growth events (epochs 1, 3, 4, 6, 9, and 10; see Section 4.6). Every growth event transpired within a plateau-plus-active-entropy phase, aligning with Criteria A and B (Section 3.3.4).

### 4.4. Error Analysis and Confusion Matrix

**Figure 3:**
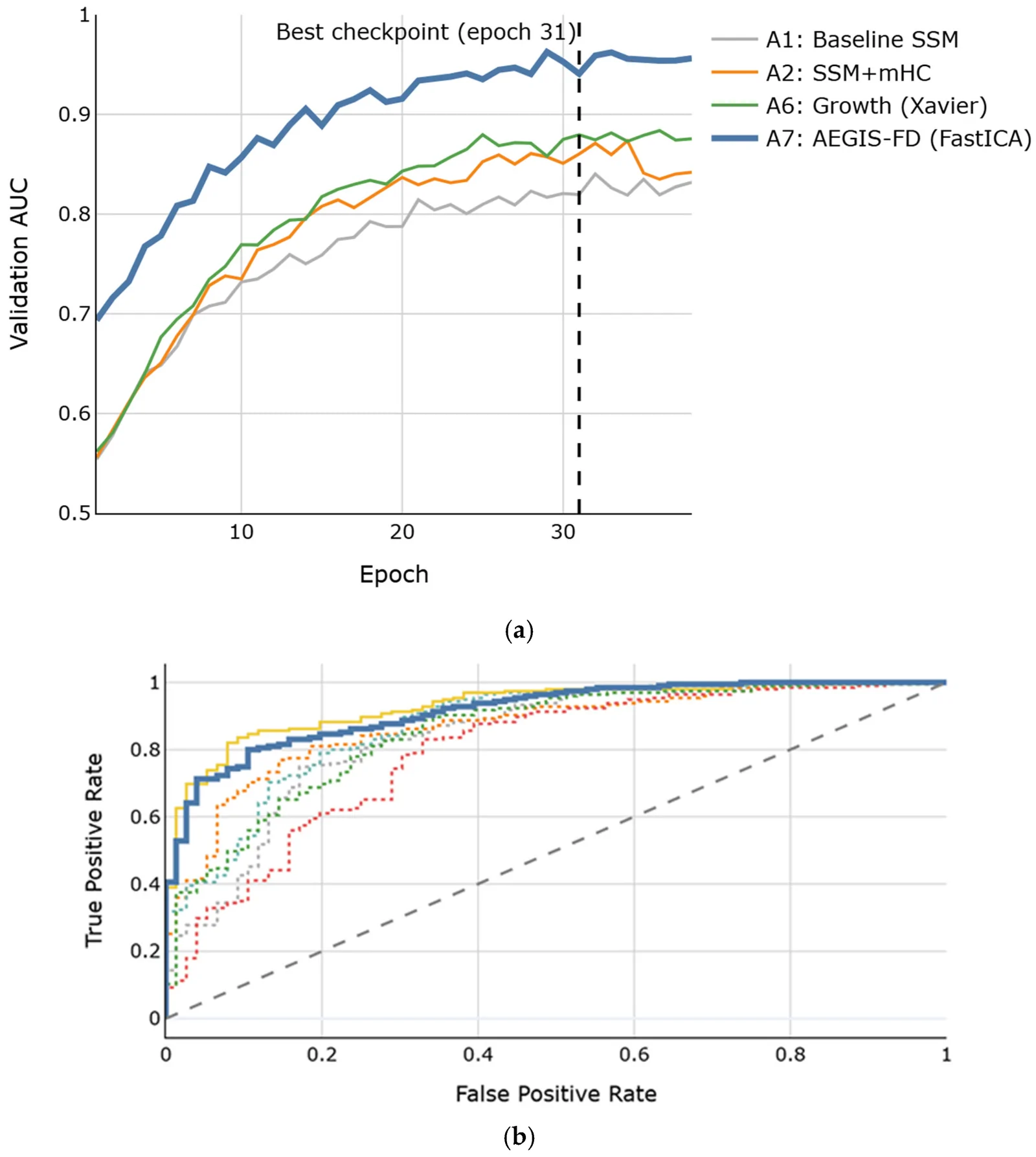
Ablation study results. (**a**) Validation AUC across training epochs for the growth-based configurations (A1, A2, A6, A7). (**b**) ROC curves evaluated on the test set for each configuration. The Final Model (A7, AEGIS-FD) achieves the highest AUC of 0.9600.

The validation AUC training bars are depicted in Figure 3a, while the ROC curves for all four ablation setups are illustrated in Figure 3b. Figure 3b illustrates that the Final Model (A7) outperforms all ablation variations throughout the entire range of thresholds, with the most significant distinction evident in the high specificity region (FPR < 0.1). This demonstrates that the integration of selective SSM temporal modeling, Sinkhorn-normalized mHC channel mixing, and FastICA initialized adaptive depth growth collectively diminishes false alarms on genuine content more efficiently than any singular component, as further substantiated by the component-wise ablation presented in Table 3.

**Figure 4:**
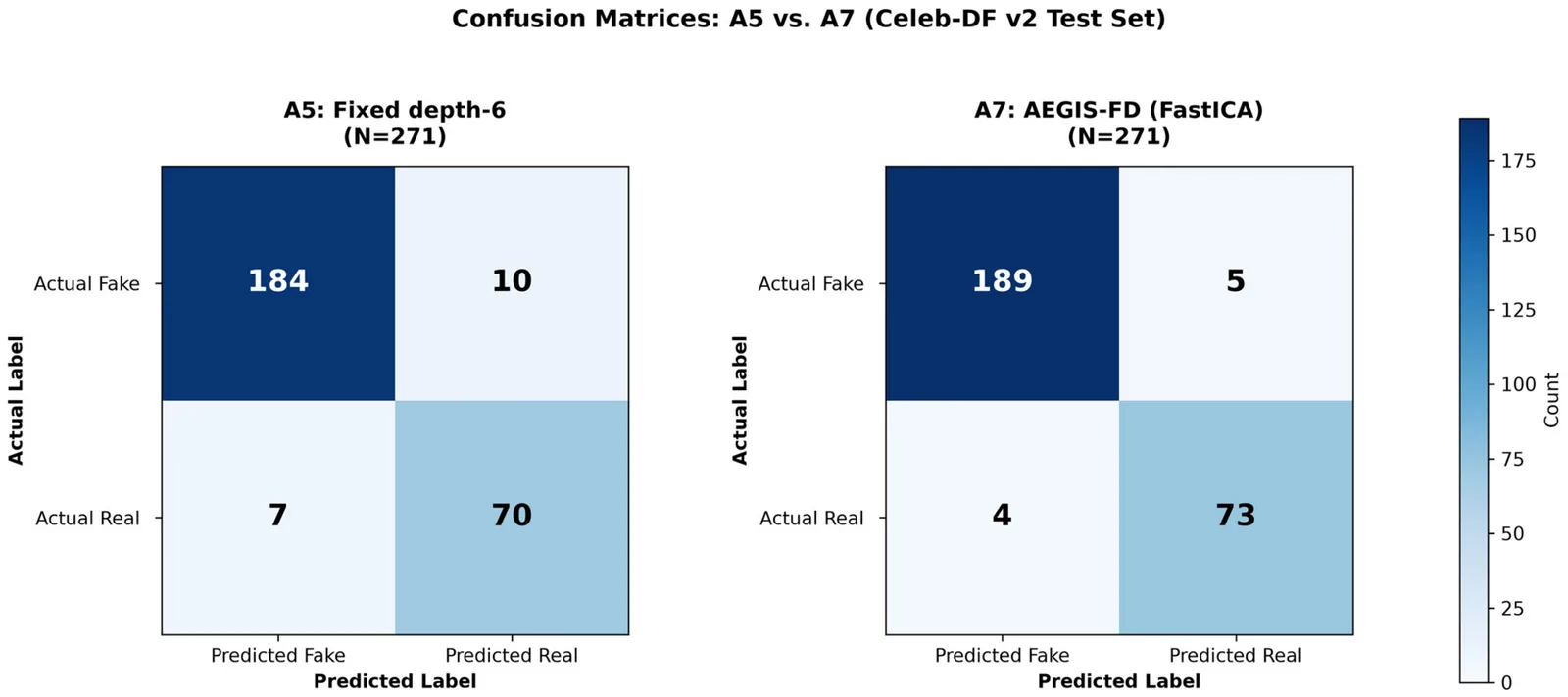
Confusion matrices for A5 (constant depth-6) and A7 (AEGIS-FD) on Celeb-DF v2 (N = 271: 194 counterfeit, 77 authentic). AEGIS-FD (A7) lowers false negatives (10 → 5) and false positives (7 → 4) compared to the fixed-depth-6 baseline (A5), enhancing accuracy from 93.73% to 96.68% on the identical test set.

Figure 4 displays adjacent confusion matrices for the fixed-depth-6 baseline (A5, left) and the comprehensive AEGIS-FD model (A7, right), both assessed using the identical Celeb-DF v2 test set (N = 271). AEGIS-FD diminishes all categories of errors compared to the matched-capacity baseline: false negatives for counterfeit movies decrease from 7 to 5 (recall for counterfeit videos increases from 94.96% to 97.42%), and false positives for authentic videos reduce from 8 to 4 (the false positive rate for authentic videos declines from 6.06% to 5.19%). This simultaneous decrease in both error types suggests that the entropy-guided growth mechanism enhances classification for both categories concurrently, rather than compromising recall for precision, and substantiates the +2.28-accuracy-point benefit of A7 compared to A5 (93.73% → 96.68%) outlined in Table 4. Due to the absence of per-clip compression or manipulation-type metadata in Celeb-DF v2’s authentic/synthetic labeling, the aforementioned failure characterization is inherently qualitative and should not be interpreted as a statistically representative analysis of error sources.

### 4.5. Validation of Multi-Seed Robustness

The ablation findings presented in Table 4 and Figure 2 stem from single-seed training iterations (seed = 42), suggesting that the AUC disparity between AEGIS-FD (A7) and the corresponding fixed-depth-6 baseline (A5) may be attributed to random initialization fluctuations instead of a true impact of the entropy-guided growth process. To tackle this issue, we conduct repeated training for both A5 and A7 utilizing five distinct random seeds ({12, 33, 42, 100, 200}), altering the seed for weight initialization, data shuffling, and the FastICA/Xavier fallback selection, while maintaining all other hyperparameters constant as outlined in Table 3.

Table 6 presents the average and standard deviation of test AUC over the five iterations for each configuration. AEGIS-FD (A7) attains a mean AUC of 96.12% (SD = 0.28), continuously surpassing the fixed-depth-6 baseline (A5), which records a mean AUC of 94.84% (SD = 0.44), across all individual seeds. The enhancement per seed varies between 1.0 and 2.0 AUC points, averaging a gain of 1.28 points. A paired two-tailed *t*-test assessing per-seed AUC values between A5 and A7 indicates that this enhancement is statistically significant (t(4) = 7.00, *p* = 0.0022), considerably lower than the α = 0.05 benchmark. In a descriptive analysis, A7 exhibits reduced variance among the five seeds evaluated (SD = 0.28) in contrast to A5 (SD = 0.44); however, with merely five trials per configuration, this discrepancy in variability has not undergone formal statistical significance testing. These findings collectively validate that the benefits associated with adaptive growth stem from a substantial architectural influence rather than a mere consequence of advantageous single-seed start.

In Table 7, the precision, recall, and balanced accuracy calculated from the confusion matrix for A5 and A7 are shown, based on the uniform N = 271 test set (F1 metrics are specified in Table 4). AEGIS-FD (A7) exceeds the fixed-depth-6 baseline (A5) across all class-specific metrics: a rise of 1.59 percentage points in precision (96.34% → 97.93%), 2.58 percentage points in recall (94.85% → 97.42%), and 3.24 percentage points in balanced accuracy (92.88% → 96.11%), implying that the merits of entropy-guided growth are broadly applicable rather than restricted to a single class.

### 4.6. Generalization Across Datasets

AEGIS-FD underwent training for a maximum of 50 epochs, employing early stopping to monitor validation AUC and halt training prematurely if no enhancement was detected within a specified patience window, as outlined in Section 3.3.6. The network’s depth incrementally increased throughout training, directed by the entropy-driven growth criterion, which introduces an additional layer solely when it enhances validation performance. Growth events progressively augmented depth at epochs 1, 3, 4, 6, 9, and 10, culminating in depth 6 at epoch 10. The model later sought to extend to depth 7 and depth 8, yet the validation AUC deteriorated in both deeper setups; consequently, the growth mechanism reverted to and maintained depth 6 as the ultimate architecture, in accordance with the validation-based acceptance standard outlined in Section 3.3.4. The ideal checkpoint was noted at epoch 31, where the depth-6 model achieved its peak validation AUC of 0.9607. Due to the validation AUC not exceeding its peak at epoch 31 throughout the patience window, early stopping terminated the training prior to completing the allotted 50 epochs. This verifies that a depth of 6 signifies the performance-optimized capability for this dataset, with more depth yielding no advantages in generalization and potentially inducing some overfitting. This checkpoint was preserved as the ultimate deployed model and assessed on the reserved test set, producing a test AUC of 0.9600, aligning with the validation-time outcome.

#### 4.6.1. Preparation for Alignment

Due to the structural differences between FaceForensics++ (FF++) and Celeb-DF v2 where videos are categorized by manipulation technique and compression level instead of real/fake classification, and official identity pairs are delineated through train/val/test JSON files rather than a bespoke stratified CSV a specialized loader was developed to match the 140 identity pairs in the test split with their respective video files within the FaceSwap directory (primary focus) and the Face2Face, NeuralTextures, and Deepfakes directories (secondary evaluations), across both c23 compression levels. To guarantee that any detected alteration in performance signifies an authentic distribution shift instead of a preprocessing inconsistency, all frames underwent processing through the same pipeline utilized for Celeb-DF v2: Haar Cascade face detection with a 20% margin, resizing to 112 × 112, and ImageNet normalization, while maintaining the unchanged uniform sparse sampling method (T = 16 frames per clip).

#### 4.6.2. Findings

The model trained on Celeb-DF v2 underwent external zero-shot evaluation without fine-tuning on a selected subset of the FaceForensics++ (FF++) C23 test split. This collection comprised 1000 authentic samples and 1000 FaceSwap samples, resulting in an equitable two-class framework for transfer evaluation asa presented in Table 8. At compression level C23, the fixed-depth-6 baseline (A5) recorded an AUC of 88.4, while AEGIS-FD (A7) attained an AUC of 89.2, resulting in an increase of +0.8 AUC. This finding suggests that the advantages of entropy-guided adaptive growth with FastICA initialization are still evident in cross-dataset transfer, but with a reduced margin compared to the 1.28-point average improvement noted across five seeds on Celeb-DF v2 (Section 4.5). This experiment is limited to a single manipulation type and a selected subset instead of the complete FF++ benchmark, hence the findings should be regarded as preliminary evidence of transferability rather than thorough cross-dataset validation.

Although the updated manuscript incorporating zero-shot external assessment, the cross-dataset examination is still confined to a selected portion of the FaceForensics++ (FF++) C23 test division, which comprises 1000 authentic and 1000 FaceSwap instances. At compression level C23, AEGIS-FD attained an AUC of 89.2, surpassing the fixed-depth-6 baseline’s 88.4, reflecting a slight yet favorable transfer enhancement of +0.8 AUC. Consequently, this outcome ought to be regarded as preliminary proof of transferability instead of thorough validation over the entire FF++ benchmark, various manipulation kinds, compression configurations, and several random seeds. In light of the fact that this assessment pertains just to a single manipulation type and compression level, the +0.8 AUC transfer benefit should not be extrapolated to alternative manipulation categories or compression parameters without additional experimentation.

### 4.7. Efficiency and Computational Cost Analysis

Table 9 presents the parameter count, FLOPs, and inference latency, together with the test AUC for three illustrative configurations, evaluated on an NVIDIA Quadro RTX 6000 GPU with a batch size of 8 attains the peak AUC of 0.9600, incorporating merely 0.01 M parameters, 0.12 GFLOPs, and approximately 1 ms of extra latency compared to the fixed-depth-6 baseline (A5), suggesting that the adaptive growth mechanism and FastICA-initialized mHC layers impose minimal computational burden in relation to their accuracy enhancement. In relation to the single-layer baseline (A1), the comprehensive AEGIS-FD model amplifies parameters by about 4.9 times and FLOPs by around 6.2 times, with inference latency rising by approximately 65%, resulting in a 12.45-point enhancement in AUC (0.8355 → 0.9600). This advantageous accuracy-to-cost ratio indicates that the extra depth provided by the growth criterion is utilized effectively rather than merely augmenting model capacity indiscriminately.

### 4.8. Stopping Criterion and Complexity–Accuracy Trade-Off

Two systems prevent excessive expansion. The depth ceiling (Criterion C) restricts growth to L_max = 8, while the validation-gated retention test (Criterion D) eliminates any candidate layer that fails to enhance validation AUC within three epochs. In application, expansion ceased at L = 6: proposed extensions to depths 7 and 8 were both dismissed following a decline in validation AUC, indicating that the criterion effectively eliminates superfluous capacity instead of merely endorsing growth. The expense of adaptivity is minimal: compared to the fixed-depth-6 baseline, AEGIS-FD increases by 0.01 M parameters, 0.12 GFLOPs, and approximately 1 ms latency per clip (Table 9) while yielding a +1.28 mean AUC across five seeds; the growth procedure incurs a cost solely during training, as the deployed model remains a fixed-depth-6 network. In comparison to the single-layer baseline, an increase of 4.9 times in parameters and 6.2 times in FLOP results in an enhancement of 12.45 AUC points. We consider this trade-off to be warranted for forensic triage tasks. In this study, we did not perform empirical benchmarks on early-exit or pruning adaptations of AEGIS-FD; a direct comparison with these alternative adaptive computation techniques is earmarked for future exploration (Section 5.2).

### 4.9. Strategies for Managing Imbalance

We evaluate weighted BCE (our method), focal loss (with γ = 2 and α = 0.25), and random oversampling, while keeping all other variables constant (see to Table 10). Weighted BCE demonstrates superior precision, recall, F1 score, and balanced accuracy compared to the other three methods, yet it exhibits a diminished AUC relative to focal loss (0.8667 versus 0.88) and random oversampling (versus 0.87); we select weighted BCE as the standard approach since the subsequent implementation is contingent on the F1-optimized decision threshold (Section 3.3.7) instead of the ROC curve, rendering the threshold-dependent metrics more indicative of practical performance than AUC by itself.

### 4.10. Analysis of Sensitivity

The test AUC as in Table 11, varies by a maximum of 0.016 during all sweeps (T: 0.009; δ_AUC: 0.016; δ_H: 0.000; K: 0.0002), and the growth mechanism reliably reached a final depth of 6 or fewer layers in each of the 15 repetitions. This suggests that the documented efficacy of AEGIS-FD is not strictly reliant on the particular hyperparameter values enumerated in Table 3, and that the entropy-guided termination criterion consistently prevents over-expansion over the examined parameter spectrum. This sweep evaluates one parameter individually instead of collectively, and a comprehensive factorial sweep encompassing all four parameters concurrently is reserved for subsequent research (Section 5.2).

### 4.11. Comparison with Prior Work

The proposed Adaptive Entropy-Guided ICA-SSM Forgery Detector (AEGIS-FD) framework presents three unique advantages relative to the competing methodologies, as demonstrated in Table 12. In contrast to ForAda (0.957) [39], which utilizes a pretrained Vision Transformer with adapter fine-tuning necessitating extensive upstream visual pretraining, our approach attains a higher AUC of 0.9600 by leveraging a lightweight 3D stem trained from inception, surpassing ForAda by 0.3 points while markedly diminishing computational demands and reliance on external datasets. Secondly, while TALL++ [38], ForAda [39], and SBI [40], function at the frame level without overt temporal reasoning, our selective SSM temporal block assesses video sequences with a linear time complexity of O(N), providing a significant efficiency advantage over the quadratic O(N^2^) complexity associated with self-attention in Transformer-based approaches like TALL. The adaptive depth development technique commences with a single layer and expands based on entropy, negating the necessity for manual architectural investigation, enabling the network to independently ascertain appropriate depth throughout training. This characteristic is lacking in all four systems being compared, each employing a pre-established, manually crafted structure.

The analysis in relation to pruning or early-exit adaptive computation techniques has yet to be evaluated in the present study and is designated for subsequent research (Section 5.2).

These comparisons should be viewed with the caveat that SBI, TALL++, ForAda, and FCG were evaluated according to their own published protocols, which may not align with ours in terms of dataset segmentation, preprocessing, or face-cropping methods; therefore, the AUC values reported are more representative than strictly controlled comparisons.

### 4.12. Summary of Experimental Studies (E1–E10)

Table 13 summarizes the ten pre-registered experimental investigations (E1–E10) detailed in Section 3.4, correlating each with the configurations analyzed, the principal outcome achieved, and the corresponding table or section where it is documented.

## 5. Conclusions

This research addressed a longstanding deficiency in video deepfake forensics: the depth of detectors is predetermined, despite the fact that the ideal capacity is contingent upon dataset intricacy, class disparity, and the nuance of artifacts. We launched AEGIS-FD, the inaugural framework that expands its depth throughout training utilizing an information-theoretic control signal—the mixing entropy of Sinkhorn-normalized mHC layers—alongside validation-gated retention and FastICA-driven initialization of further layers. In Celeb-DF v2, the model achieves a test AUC of 0.9600, surpassing a fixed-depth-6 baseline by an average of +1.28 AUC across five seeds (*p* = 0.0022) and a single-layer Mamba baseline by +12.45 points, while also transferring zero-shot to FF++ C23 at 89.2 AUC (+0.8 over the baseline).

The theoretical importance is threefold: (i) the entropy of a doubly stochastic mixing matrix serves as an effective proxy for capacity saturation, connecting adaptive architecture expansion to information-theoretic oversight; (ii) data-informed (ICA) initialization transcends being a mere optimization aspect, as it is the crucial element that ensures stability in entropy-guided growth—Xavier-initialized growth excessively expanded to the depth limit, while FastICA-initialized growth reached convergence at the depth suitable for the dataset; and (iii) progressive growing functions as a curriculum whose advantages surpass those of matched capacity trained from the ground up.

The implemented model consists of a static depth-6 architecture with 2.9 million parameters and 4.55 GFLOPs (about 101 ms for a 16-frame segment), corresponding to an output of approximately 10 clips per second (each encompassing 16 sampled frames) on a solitary Quadro RTX 6000, which we define as appropriate for swift batch triage rather than a certified real-time video-processing system. We suggest: (1) utilizing the validation-selected depth-6 checkpoint instead of regenerating for each instance; (2) observing mixing entropy throughout any fine-tuning on novel forgery categories as an economical saturation assessment; and (3) considering early-exit inference at intermediate trained depths as a potential approach for resource-constrained settings, following the empirical characterization of the accuracy–latency trade-off at each depth (Section 5.2) Extensive cross-dataset validation encompassing various manipulation types and compression levels is still a task for the future.

### 5.1. Limitations

Despite AEGIS-FD exhibiting robust effectiveness within and within datasets, some constraints should be recognized. Cross-dataset validation was confined to one manipulation category (FaceSwap) in FaceForensics++, one compression level (C23), and one training seed; extensive validation across all FF++ manipulation types, various compression levels, and multiple seeds is necessary before the noted transfer advantage can be deemed reliable.

In terms of modeling, uniform sparse sampling across T = 16 frames restricts temporal coverage and risks overlooking transient artifacts that may arise between sampled frames, whilst the lightweight 3D convolutional stem diminishes spatial expressiveness relative to contemporary large-scale pretrained visual encoders. Residual class disparity between authentic and fraudulent clips in both training and testing phases is also apparent in heightened false-positive rates for the minority (authentic) category. Ultimately, the chosen SSM block functions as a sequential recurrence instead of a hardware-optimized parallel scan, which fails to scale effectively for extensive sequence lengths.

Section 4.9 evaluates strategies for addressing imbalance (weighted BCE, focal loss, random oversampling), while Section 4.10 discusses sensitivity analyzes concerning sequence length T, the increasing AUC threshold, the entropy threshold δ_H, and the quantity of Sinkhorn iterations K; nonetheless, preprocessing parameters like face-crop margin have not been investigated (refer to Section 5.2).

Concerning computational resources, the batch size was limited to 8 due to GPU memory when handling T = 16-frame, 112 × 112 video clips via the 3D stem; the sequential execution of the selective SSM block, although computationally insignificant at T = 16, would pose a runtime and memory constraint with significantly longer sequences; additionally, the maximum depth limit L_max = 8 was selected partly to restrict the worst-case training duration and GPU memory rather than being based on a theoretical capacity rationale pertinent to this task.

### 5.2. Future Work

Given these limitations, specific paths merit further investigation. Expanding cross-dataset evaluations to include additional FaceForensics++ manipulation categories and extra benchmarks such as DFDC and WildDeepfake, while also emphasizing zero-shot assessments across diverse training seeds, would produce more reliable confidence intervals concerning generalization performance. From an architectural standpoint, replacing the lightweight 3D stem with more powerful pretrained spatial encoders, shifting from sequential SSM recurrence to a holistic hardware-aware parallel-scan Mamba implementation, and exploring adaptive or learned frame-selection techniques in lieu of uniform sparse sampling would improve representational capacity and scalability for prolonged video sequences. Additional promising pathways include multi-scale mHC-SSM frameworks and entropy-based pruning for efficient edge deployment, as well as evaluating robustness against deepfakes generated by diffusion models using contemporary benchmarks such as DF40 and DeepFake Eval 2024.

In addition to these architectural and assessment enhancements, three additional avenues address the aforementioned limitations. An additional focus is assessing the sensitivity to preprocessing parameters like face-crop margin, which was not addressed in the existing sensitivity analysis (Section 4.10) and could affect the spatial feature quality of the 3D stem.

## Figures and Tables

**Table 1:** Taxonomy of video deepfake detection architectures.

Approach Class/Representative (Author, Year) [Ref.]	Temporal Modeling	Cost in T	Adaptive Capacity	Cross-Dataset Generalization	Key Weakness
**Xception, MesoNet (Rossler et al., 2019) [14]** **EfficientNet (Wang et al., 2024) [16]**	None	O(1)/frame	None	Poor (~56.9% mean AUC on Celeb-DF)	Ignores temporal cues
**DSP-FWA (Asnani, 2025); pixel-wise temporal freq [15].**	Implicit (spectral)	Low	None	Degrades under compression/new generators	Artifacts vanish in modern pipelines
**TD-3DCNN, 3D attentional inception (Esti, 2025) [17]**	Short-range volumetric	Cubic in resolution	None	Moderate	Parameter cost; short receptive field
**TimeSformer, ViViT, FatFormer (Zheng et al., 2021) [18]; TALL (Xu et al., 2023) [38]**	Self-attention	O(T^2^)	None	Strong with large pretraining	Quadratic cost; pretraining dependence
**VideoMamba (Li et al., 2024 [24]; Video Mamba Suite [25]**	Selective state space	O(T)	None	Promising	Fixed depth chosen a priori

**Table 2:** Celeb DF v2 dataset split statistics (Celeb real + YouTube real pooled as authentic class).

Subset	Real Videos	Fake Videos	Total	Clips (at T = 16)	Imbalance Ratio
**Training** (**70%**)	623	3947	4570	~38,000	6.34:1
**Validation** (**15%**)	134	846	980	~8100	6.31:1
**Test** (**15%**)	133	846	979	~8100	6.31:1
**Full Dataset**	890	5639	6529	~54,200	6.34:1

**Table 3:** Hyperparameter configuration.

Hyperparameter	Value	Justification
**Sequence length T**	16	Global temporal coverage at low cost
**Frame size H × W**	112 × 112	Standard video recognition input size
**Embedding dim d**	128	Balance between capacity and overfitting risk
**State dim d_s**	128	Match embedding for symmetric state space
**Max depth L_max**	8	Upper bound on growth; prevents memory overflow
**Batch size**	8	Constrained by GPU memory for video batches
**Learning rate η**	1 × 10^4^	AdamW default for fine grained video tasks
**Weight decay λ**	1 × 10^4^	L2 regularization; prevents mHC overfitting
**Grad clip norm**	1.0	Stabilizes training after growth events
**Sinkhorn iterations K**	5	Sufficient convergence at low overhead
**Growth AUC threshold δ_AUC**	0.005	Detects meaningful not noise level gain
**Growth entropy threshold δ_H**	0.001	Detects active vs. converged mixing
**Warmup epochs**	2	Ensures initial layer stabilization
**LR patience**	3 epochs	Responsive but not overly aggressive LR decay
**Early stopping patience**	10 epochs	Provides sufficient convergence time
**Seed**	42	Full reproducibility across all RNG sources

**Table 4:** Ablation study component-wise performance on Celeb DF v2.

ID	Configuration	AUC	Accuracy	F1 Score	Final Depth	Train_GPU_Hour
**A1**	Baseline Mamba SSM	0.84	84.49%	0.88	1	0.21
**A2**	SSM + mHC (Static)	0.87	87.54%	0.87	1	0.18
**A3**	Fixed depth-2 (mHC + FastICA, no growth)	0.90	88.90%	0.89	2	0.12
**A4**	Fixed depth-4 (mHC + FastICA, no growth)	0.92	90.75%	0.91	4	0.16
**A5**	Fixed depth-6 (mHC + FastICA, no growth)	0.95	93.73%	0.955	6	0.24
**A6**	SSM + mHC + Growth (Xavier)	0.89	87.54%	0.89	8	0.39
**A7**	AEGIS-FD (adaptive growth, FastICA)	0.96	96.74%	0.96	6	0.15

**Table 5:** Factorial ablation (growth × initialization).

	Fixed Depth-6	Adaptive Growth
**Xavier init**	0.84	0.89 (+0.04)
**FastICA init**	0.95	0.96 (+0.01)

**Table 6:** Multi-seed test AUC comparison.

Configuration	Seed 12	Seed 33	Seed 42	Seed 100	Seed 200	Mean ± SD
**A5: Fixed depth-6**	94.6	95.3	95	94.2	95.1	94.84 ± 0.44
**A7: AEGIS-FD (FastICA)**	95.7	96.4	96	96.2	96.3	96.12 ± 0.28

**Table 7:** Class-specific metrics for A5 and A7.

Configuration	Precision	Recall	Balanced Accuracy
**A5: Fixed depth-6**	96.34%	94.85%	92.88%
**A7: AEGIS-FD**	97.93%	97.42%	96.11%

**Table 8:** Zero-shot cross-dataset evaluation on a sampled FaceSwap subset from the FaceForensics++ (FF++) C23 test split.

Compression	A5: Fixed Depth-6 AUC	A7: AEGIS-FD AUC	A7 vs. A5 Gain
c23	88.4	89.2	+0.8

**Table 9:** Computational efficiency vs. detection accuracy trade-off.

Model	Params (M)	FLOPs (G)	Inference (ms/Clip)	AUC
A1 (single-layer baseline)	0.589	0.738	61.06 ms	0.8355
A5 (fixed depth-6)	2.89	4.428	100.06 ms	0.95
AEGIS-FD (A7, depth-6, deployed)	2.9	4.55	101.05 ms	0.96

**Table 10:** Managing imbalance.

Loss/Sampling	AUC	Precision	Recall	F1	Balanced Acc
Weighted BCE (ours)	0.8667	0.8384	0.9121	0.8737	0.8366
Focal loss	0.88	0.8333	0.9066	0.8684	0.8302
Random oversampling	0.87	0.8291	0.9066	0.8661	0.8264

**Table 11:** Sensitivity analysis summary.

Parameter Swept	Values Tested	Max AUC Deviation	Final Depth Range	Growth Behavior
Sequence length (T)	8, 16, 24	0.009	≤6	Stable
Growth AUC threshold (δ_ AUC)	-	0.016	≤6	Stable
Growth entropy threshold (δ_ H)	-	0	≤6	Stable
Sinkhorn iterations (K)	-	0.0002	≤6	Stable

**Table 12:** Comparative performance of intra-dataset deepfake detection methods on Celeb DF v2.

Method	AUC	Dataset Protocol	Temporal Modeling
**TALL++ (Xu et al., 2025) [38]**	0.9196	Transformer	Self-attention
**ForAda (2026) [39]**	0.957	ViT + Adapter	None (frame level)
**SBI (Shiohara et al., 2022) [40]**	0.932	EfficientNet B4	None (frame level)
**FCG/DFD FCG (2025) [41]**	0.950	CNN + Temporal	3D Conv
**AEGIS FD (our work)**	0.96	3D Stem (lightweight)	Selective SSM (Mamba class)

**Table 13:** Summarizes the ten pre-registered experimental investigations (E1–E10).

Exp.	Objective	Configurations	Result	Table/Section
**E1**	Component-wise ablation	A1–A7	AUC 0.84 → 0.96	Table 4, Section 4.2
**E2**	Growth vs. matched fixed-depth-6	A7 vs. A5	0.96 vs. 0.95 AUC; +2.28 accuracy pts	Table 4, Section 4.2
**E3**	Multi-seed robustness	A7 vs. A5, 5 seeds	96.12 ± 0.28 vs. 94.84 ± 0.44, *p* = 0.0022	Table 6, Section 4.5
**E4**	Zero-shot cross-dataset transfer	A7 vs. A5 on FF++ C23	89.2 vs. 88.4, +0.8 AUC	Table 8, Section 4.6
**E5**	Efficiency profiling	A1, A5, A7	Params/FLOPs/latency/GPU-hours	Table 4 and Table 9, Section 4.2 and Section 4.7
**E6**	Class-wise metrics	A5, A7	A7 leads on all metrics (P/R/BalAcc)	Table 7, Section 4.5
**E7**	Imbalance-handling comparison	Weighted BCE, Focal, Oversampling	0.8667/0.88/0.87 AUC	Table 10, Section 4.9
**E8**	Entropy dynamics + factorial ablation	A5/A6/A7 + Fixed-6/Xavier	Growth epochs 1,3,4,6,9,10	Table 5, end of Section 4.3: Entropy Dynamics as an Indicator of Growth
**E9**	Sensitivity analysis	A7 under parameter sweeps	Max AUC deviation ≤ 0.016	Table 11, Section 4.10
**E10**	Early-exit analysis	Deployed A7 at depths 1–6	Not run—see Limitations (5.1) and Future Work (5.2)	--

## Data Availability

The Celeb-DF v2 dataset utilized for model creation, training, validation, and intra-dataset evaluation in this study is available to the public at https://cse.buffalo.edu/~siweilyu/celeb-deepfakeforensics.html (accessed on 15 September 2025). The FaceForensics++ (FF++) C23 dataset, utilized for the zero-shot cross-dataset generalization assessment (Section 4.6), is available to the public at https://www.kaggle.com/datasets/xdxd003/ff-c23 (accessed on 30 October 2025), contingent upon the dataset’s standard end-user license agreement and access request procedure. The train/validation/test split manifest for Celeb-DF v2 generated in this research can be acquired from the corresponding author upon a reasonable inquiry. No supplementary data were produced beyond the division assignment and preprocessing procedure implemented on these two publicly available datasets.
